# The metabolic, virulence and antimicrobial resistance profiles of colonising *Streptococcus pneumoniae* shift after PCV13 introduction in urban Malawi

**DOI:** 10.1038/s41467-023-43160-y

**Published:** 2023-11-17

**Authors:** Uri Obolski, Todd D. Swarthout, Akuzike Kalizang’oma, Thandie S. Mwalukomo, Jia Mun Chan, Caroline M. Weight, Comfort Brown, Rory Cave, Jen Cornick, Arox Wadson Kamng’ona, Jacquline Msefula, Giuseppe Ercoli, Jeremy S. Brown, José Lourenço, Martin C. Maiden, Neil French, Sunetra Gupta, Robert S. Heyderman

**Affiliations:** 1 Department of Epidemiology and Preventive Medicine, School of Public Health, Faculty of Medicine, Tel Aviv University, Tel Aviv, Israel; 2 Porter School of the Environment and Earth Sciences, Faculty of Exact Sciences, Tel Aviv University, Tel Aviv, Israel; 3 Malawi Liverpool Wellcome Programme, Blantyre, Malawi; 4 Mucosal Pathogens Research Group, Research Department of Infection, Division of Infection & Immunity, University College London, London, United Kingdom; 5 Julius Center for Health Sciences and Primary Care, University Medical Center Utrecht, Utrecht, Netherlands; 6 Kamuzu University of Health Sciences, Blantyre, Malawi; 7 Faculty of Health and Medicine, Biomedical and Life Sciences, Lancaster University, Lancaster, United Kingdom; 8 Biomedical and Life Sciences, Faculty of Health and Medicine, Lancaster University, Lancaster, United Kingdom; 9 Clinical Infection, Microbiology and Immunology, Institute of Infection Veterinary & Ecological Science, University of Liverpool, Liverpool, United Kingdom; 10 UCL Respiratory, Division of Medicine, University College London, London, United Kingdom; 11 Department of Zoology, University of Oxford, Oxford, United Kingdom; 12 Universidade Católica Portuguesa, Faculty of Medicine, Biomedical Research Centre, Lisbon, Portugal

**Keywords:** Bacterial genetics, Population genetics, Genomics, Epidemiology, Vaccines

## Abstract

*Streptococcus pneumoniae* causes substantial mortality among children under 5-years-old worldwide. Polysaccharide conjugate vaccines (PCVs) are highly effective at reducing vaccine serotype disease, but emergence of non-vaccine serotypes and persistent nasopharyngeal carriage threaten this success. We investigated the hypothesis that following vaccine, adapted pneumococcal genotypes emerge with the potential for vaccine escape. We genome sequenced 2804 penumococcal isolates, collected 4-8 years after introduction of PCV13 in Blantyre, Malawi. We developed a pipeline to cluster the pneumococcal population based on metabolic core genes into *“Metabolic genotypes”* (MTs). We show that *S. pneumoniae* population genetics are characterised by emergence of MTs with distinct virulence and antimicrobial resistance (AMR) profiles. Preliminary in vitro and murine experiments revealed that representative isolates from emerging MTs differed in growth, haemolytic, epithelial infection, and murine colonisation characteristics. Our results suggest that in the context of PCV13 introduction, pneumococcal population dynamics had shifted, a phenomenon that could further undermine vaccine control and promote spread of AMR.

## Abstract (web summary)

Pneumococcal vaccination has been shown to promote emergence of non-vaccine *S. pneumoniae* serotypes. Here, the authors use data from Malawi to investigate whether vaccine introduction also results in changes in metabolic, virulence, and antimicrobial resistance profiles of circulating strains.

## Introduction

*Streptococcus pneumoniae* (the pneumococcus), a common commensal of the upper respiratory tract, is responsible for a high burden of severe pneumonia, septicaemia and meningitis^1,2^, mainly affecting children under 5-years-old, the immunocompromised and the elderly. Pneumococcal disease causes almost 300,000 deaths annually in under-5’s worldwide, and disproportionally affects people in resource-poor settings, where 57% of the total pneumococcal deaths occur^3^. This high burden of pneumococcal disease is largely vaccine preventable^1,4^. Indeed, reports from The Gambia, South Africa, Kenya and Malawi^5–9^ show that pneumococcal conjugate vaccines (PCV), targeting the polysaccharide capsule of the most common disease-causing pneumococcal serotypes (currently PCV10 or PCV13), have been highly effective in reducing invasive pneumococcal disease (IPD). However, the highly adaptable nature of *S. pneumoniae* has the potential to undermine this success. Through the expression of approximately 100 capsule serotypes, the pneumococcus has the ability to adapt to change, by mutation and by the capacity to readily acquire new traits through horizontal gene transfer from other pneumococci and related streptococci occupying the same niche^10,11^.

In the context of PCV introduction, vaccine type (VT) replacement IPD is widespread in resource-rich settings^12,13^ and is being increasingly reported on the African continent^14,15^. In addition, we and others have shown that despite excellent vaccine uptake in many African countries, there is considerable residual VT nasopharyngeal carriage in both PCV-vaccinated and PCV-unvaccinated populations several years after PCV introduction^6,7,16^. Routine PCV programmes have also been shown to reduce the burden of antimicrobial resistant (AMR) *S. pneumoniae* VT disease and carriage in some settings^17,18^. However, the impact has not been uniform, with the emergence of certain AMR serotypes, most notably 7F and 19A^19–21^. Furthermore, our modelling suggests that under vaccine pressure, antimicrobial resistant non-VT (NVT) strains could replace susceptible NVT strains through the removal of competition from vaccine-susceptible VTs^22^.

Emerging evidence from Africa, Europe and the USA suggests that PCV introduction has driven genomic alterations in *S. pneumoniae* populations, contributing to capsular switching, selection of specific lineages and genetic recombination^21,23–26^. We have previously described a theoretical framework whereby pneumococcal vaccines targeting particular serotypes drive the emergence of NVT *S. pneumoniae* strains with metabolic and virulence-associated characteristics similar to the VTs commonly circulating prior to vaccine introduction^27,28^.

Here, to explore the genotypic and phenotypic basis for this framework, we have used a large, well-characterised pneumococcal carriage strain collection from an urban population in Blantyre, Malawi (2015–2019), established starting 4 years after the introduction of PCV13 into the national immunisation programme. PCV13 was introduced 12 November 2011 using a 3 + 0 schedule, with primary doses given at 6, 10 and 14 weeks of age. Field studies in Malawi have reported high PCV13 uptake of 90%–95%^29^, similar to the 92% PCV13 coverage recently reported by WHO/UNICEF (WHO and UNICEF estimates of immunisation coverage, 2018. https://www.who.int/immunisation/monitoring_surveillance/routine/coverage/WUENIC_notes.pdf). We hypothesised that in this population with a high force of infection^30^ and a high frequency of multiple serotype carriage^31,32^, there would be an increase in pneumococcal diversity, adaptability and potentially AMR. We have therefore developed a metabolic core genome allelic profiling method, which has allowed us to analyse 2804*S. pneumoniae* carriage isolates collected over 4 years of surveillance. We show that discreet “metabolic genotypes” have expanded amongst both VT and NVT serotypes up to 7 years after vaccine introduction. These expanding metabolic genotypes (MT) are characterised by accessory genes linked to virulence and AMR. Strains selected from the emerging MT have in vitro and in-vivo phenotypes that suggest adaptation. Together these data highlight the genetic changes in pneumococcal population structure and biology beyond serotype replacement that, if not addressed, may undermine current and future vaccine strategies.

## Results

### Homogeneity of the Blantyre pneumococcal carriage strain collection across age-cohorts using classical serotyping and genotyping methods

**Fig. 1:**
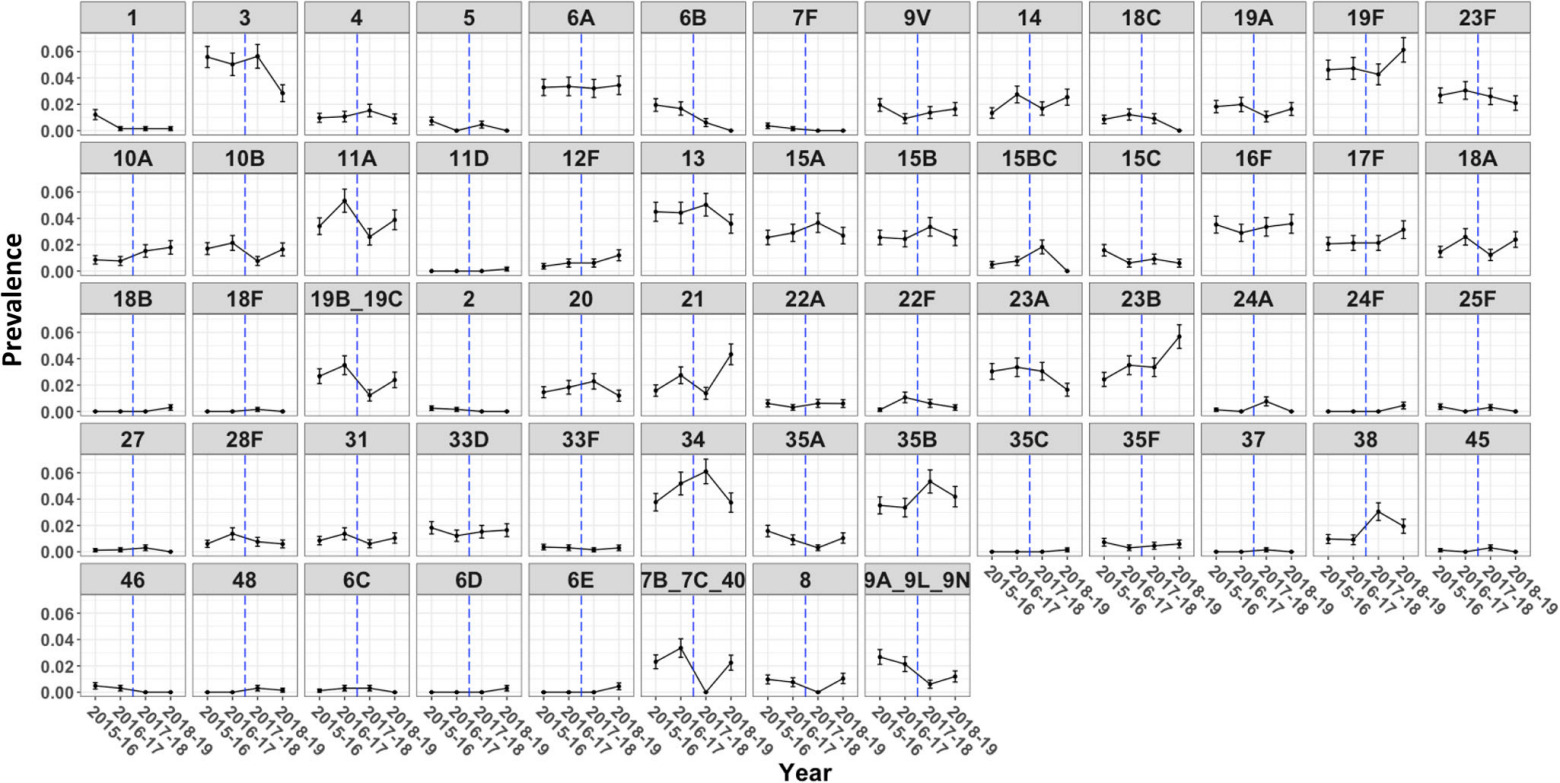
Prevalence of *S. pneumoniae* serotypes identified. The upper row shows prevalence (fraction) of vaccine serotypes (1–23F). The following rows show non-vaccine serotypes. Non-typeable strains are excluded from the analysis. The dashed blue line separates early-stages of carriage surveys (June 2015–April 2017, survey 1–4) from late stages of carriage surveys (May 2017–June 2019, survey 5–8). Serotypes are determined by WGS using the PneumoCaT tool. Error bars show standard error of the mean. The number of isolates for each point is given in Table S3.

We have previously reported an analysis of the first 7 surveys from our population-based carriage surveillance in Blantyre, Malawi (2015–2018), which showed high persistent residual VT carriage among PCV-vaccinated children 3–5-year-old (16·7%) and PCV-unvaccinated children 6–8-year-old (15·7%) and HIV-infected adults 18–40 years old on antiretroviral therapy (ART; 8·9%)^16^. Including swabs that were collected from an 8th survey conducted in 2019, overall (NT + NVT) *S. pneumoniae* carriage prevalence was 5115/8413 (60.8%). Using this Blantyre pneumococcal carriage strain collection (Table 1), WGS libraries were developed using a random subset of isolates from each of the eight surveys (Fig. 1).

**Table 1:** Number of isolates sequenced per community carriage survey

		PCV-vaccinated children (2–7 yrs)^b^		PCV-unvaccinated children (5–10yrs)		Adults (18–40 yrs HIV+ on ART)	
Survey	Survey dates	Carriage prevalence (%)^a^	No. of isolates sequenced	Carriage prevalence (%)^a^	No. of isolates sequenced	Carriage prevalence (%)^a^	No. of isolates sequenced
1	June 2015 – August 2015	241/286 (84.3)	234	174/255 (68.2)	137	78/198 (39.4)	75
2	October 2015–April 2016	230/303 (75.9)	203	144/231 (62.3)	128	95/201 (47.3)	71
3	May 2016–October 2016	282/361 (78.1)	247	142/242 (58.7)	114	124/279 (44.4)	0
4	November 2016–April 2017	351/504 (69.6)	227	75/198 (37.9)	75	132/308 (42.9)	0
5	May 2017–October 2017	402/496 (81.1)	234	62/106 (58.5)	46	117/305 (38.4)	68
6	November 2017–June 2018	390/610 (63.9)	220	84/173 (48.6)	62	90/277 (32.5)	53
7	June 2018–December 2018	390/504 (77.4)	255	113/197 (57.4)	81	78/202 (38.6)	0
8	January 2019–August 2019	401/588 (68.2)	273	92/191 (48.2)	68	70/213 (32.9)	0

*No*. number, *yrs* years old, *PCV* pneumococcal conjugate vaccine.

^a^Total carriage = all *S. pneumoniae* isolates (VT, NVT and NT).

Surveys were conducted every 6 months, commencing in June 2015. Columns show the carriage prevalence in each of the 3 cohorts included in this study, and the number of sequenced isolates.

^b^There was no recruitment of children aged 2-years during surveys 1–3.

Among the final dataset of 2804 sequenced carriage isolates, we observed a decline of VT serotype 1 and 6B carriage isolates, and an increase in frequency amongst several NVTs over time, in particular 23B and 38 (Fig. 1). However, the majority of VTs and NVTs persisted over time and although there were some differences between age groups, each cohort contributed a similar proportion of the dataset at each timepoint (Figs. 1, S1). Two hundred sequence types (ST) were identified on the basis of the allelic profiles of a standard set of seven housekeeping genes (see “Methods”). Most STs were present in each cohort (Fig. S1a), with the exception of the STs circulating at very low frequencies, including ST347, ST4084, ST10880 and ST10992, which were not identified among the isolates from the HIV-infected adults. Analogously, identified serotypes and global pneumococcal sequencing clusters (GPSCs) contributed a similar proportion of the dataset at each timepoint (Fig. S1b, c). Given this spread of serotyping and genotyping characteristics throughout the collection, all isolates were aggregated into a single dataset for the subsequent analyses.

### Serotype switching amongst both vaccine and non-vaccine serotypes

On the basis of the sequence-typing, we identified 44 separate serotype switching events in the Blantyre collection, described by a total of 22 STs which corresponded to more than one serotype (Table S1). Directionality of these switch events was determined by considering the genetic relatedness of the strains and assuming that the most common serotype (capsule type in a dominant lineage) within an ST was the serotype from which the switch originated^33^. Rather than a clear shift from VTs towards NVTs, with only a few statistically significant trends found (Tables S2 and S3), low-frequency VT or NVT variants were identified to undergo switching throughout the carriage surveys in several ST and GPSC lineages (Fig. S2a, b). These data support the emerging evidence that even in the context of vaccine introduction, the process of serotype switching is not limited to VTs (e.g., ST7653 6C/6D or ST3214 35A/11A). We suggest that although a serotype switch may be directly vaccine-induced, switched serotypes may have occurred stochastically and then emerged alongside other vaccine-associated changes in population structure. Indeed, it seems likely that selection forces that are more complex than serotype-directed vaccine pressure alone facilitate many of such switches such as environmental and antimicrobial pressures^34^.

### Shifts in metabolic gene profiles of the pneumococcal population

Bacterial metabolism is intimately linked to virulence^27,35,36^. To further identify dynamic changes in the pneumococcal population structure beyond serotype switching and genotypes defined by evolutionary history alone, we defined metabolic genotypes (MTs) based on our previously described theoretical framework^27^. Each MT was classified on the basis of differences in core genes involved in metabolism and energy production, following functional annotation of the core genome of the pneumococcal population. This typing method therefore relies on sequence differences between genes involved in bacterial metabolism (i.e., function), rather than lineages defined exclusively by bacterial evolutionary history.

**Fig. 2:**
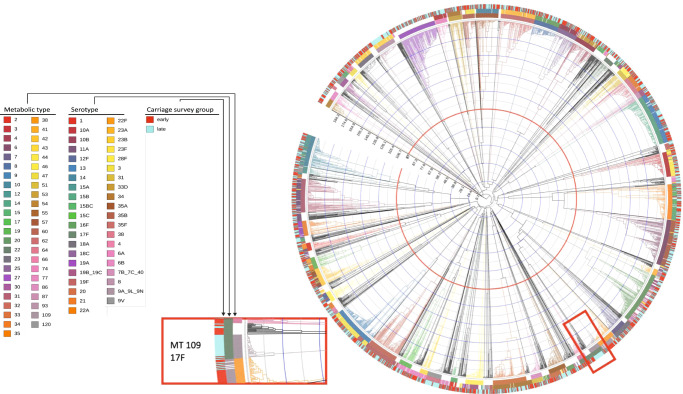
Hierarchical clustering dendrogram of *S.pneumoniae* metabolic types amongst carriage isolates (June 2015–June 2019). The dendrogram is based on of the hamming distance between the different allelic profiles of the metabolic genes. Coloured strips represent the Metabolic type, the serotype, and the carriage survey group (early = survey 1 to 4, late = survey 5 to 8). MTs are defined by curtailing the dendrogram at half the maximum distance, indicated by the red line (*n* = 148 discrete MTs, containing only MTs with more than 15 observations). The inset show a magnification of the dendrogram around serotype 17F/MT109.

We grouped the Blantyre pneumococcal carriage isolates into 148 discrete MTs (Fig. 2). As shown in the clustering tree, these MTs were neither independent of serotype nor did they completely overlap with serotype. For instance, the 14 different serotype 19F MTs were spread across the clustering tree. As might be anticipated, overlap between MTs and GPSCs has been also observed (Supplementary Data 3). Our previous study of PCV13 uptake in rural Malawi in the 1st year after vaccine introduction^29^ and our estimates of vaccine uptake in the Blantyre population 4 years after introduction^16^, show that vaccine uptake has been rapid and sustained. Our statistical modelling of the carriage survey data estimated that the population-level half-life of VT vs. NVT carriage was similar among PCV-vaccinated (3.34 vs. 9.46 years) and PCV-unvaccinated (3.26 vs. 9.83 years) children^16^. Therefore, to assess whether there had been a change in the *S. pneumoniae* genotypic profile over the surveys with sufficient precision, we compared surveys 1–4 (19 June 2015–12 April 2017) with 5–8 (3 May 2017–9 August 2019). Overall, the majority of MTs did not significantly change in frequency when comparing early and later surveillance isolates (*p* > 0·05; Fisher’s exact test), highlighting a high residual diversity within the bacterial population (Fig. S3a, b). Nonetheless, our analysis highlighted a shift in the pattern of several MTs: for instance, with the NVTs 10A, 17F, 34, 38 and 23B which showed a significant increase in MTs 74, 109, 33, 120 and 93 (Fig. S3a, b, *p* < 0·0001 for MTs 109, 120, and 93; *p* = 0.026 MT33; *p* = 0.0051 MT74 Fisher’s exact test).

In total, we observed 32 instances in which VTs switch capsular serotype to NVTs, or vice versa, while maintaining the same metabolic profile, which is in line with a vaccine-induced metabolic shift as described in ref. ^27^.

### Metabolic genotypes increasing in frequency are characterised by increased AMR

To address the hypothesis that vaccination drives changes in the pattern of AMR by removing the competition of non-AMR VTs^22^, we assessed whether the emerging MTs amongst the NVTs 10 A, 17 F, 23B, 34 and 38 were also associated with increased AMR. We determined resistance genetically to penicillin, an important first-line antibiotic for pneumococcal disease, together with chloramphenicol, erythromycin and tetracycline, which are also commonly used antibiotics in this population. Higher penicillin MICs (minimum inhibitory concentration) were observed in the later surveillance period compared to the early surveillance period (Wilcoxon rank sum test, *p* < 0·0001) (Fig. S3e). The majority of the serotypes and MTs identified in this dataset (*n* = 39 and *n* = 79, respectively) were statistically significantly associated, either positively or negatively, association with penicillin resistance (i.e., MIC > 0·06 µg/ml, *p* < 0·05; Fisher’s exact test), rather than being independent of resistance. This indicates that the phenotypic characteristic of AMR is likely linked to the isolate’s genotype^23^ (Fig. S3c, d).

**Fig. 3:**
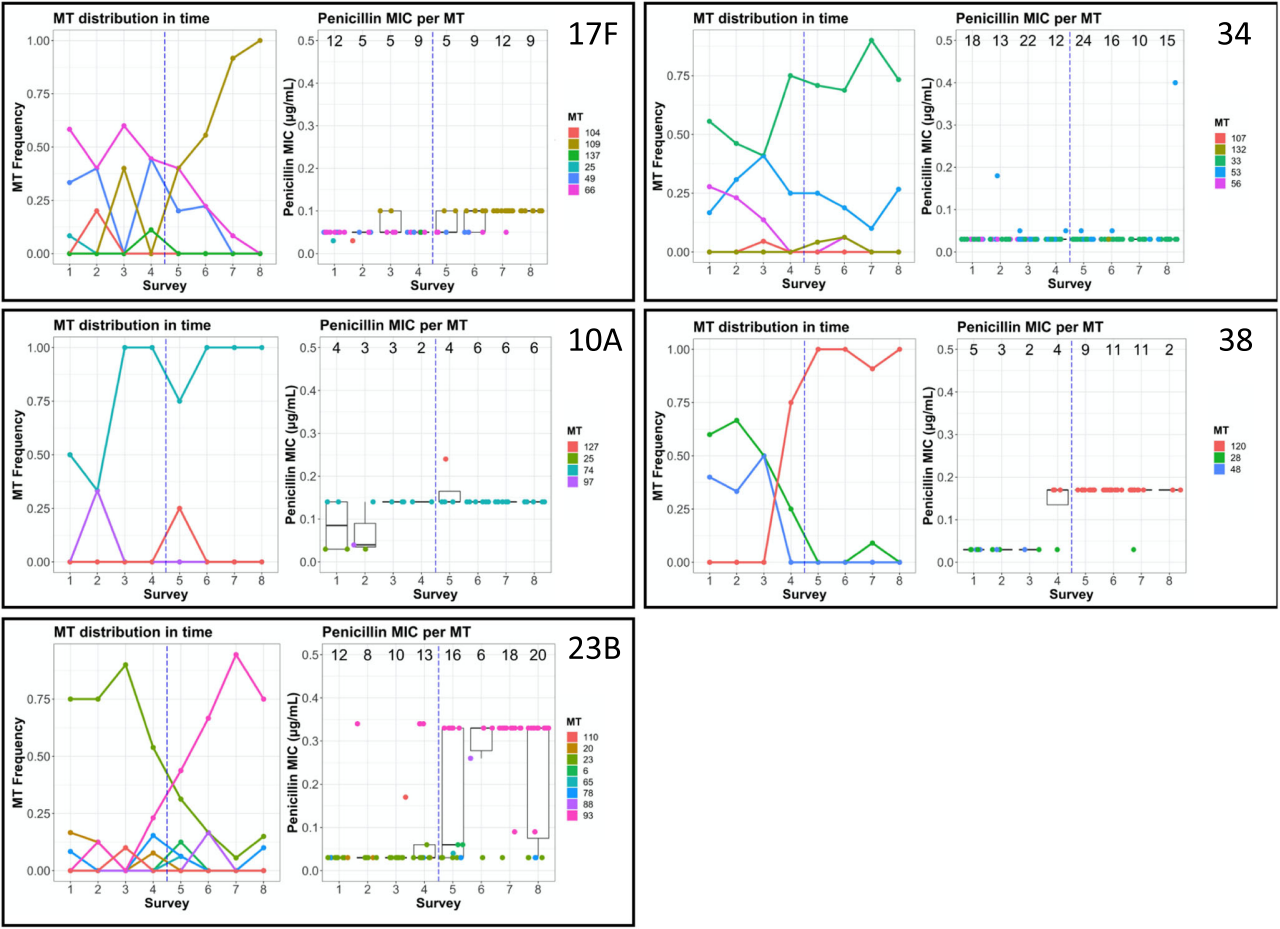
Shifts in metabolic profiles and penicillin MIC over time (surveys 1–8; June 2015–June 2019). Boxes show the frequency of isolation of each metabolic profile per survey (“MT distribution in time”) and penicillin MIC of each isolate (box plot representing the lower and upper quartiles as the box edges, a horizontal line as the median, and whiskers are the minimum between 1.5-fold the interquartile range and the farthest observation from the quartiles; with points representing isolates; and numbers on top representing the number of isolates in each survey) for to serotypes 17F, 10A, 23B, 34 and 38. Vertical blue lines separate the early-late survey periods. Presence of AMR genes per serotype is shown in Fig. S4.

We observed several instances in which emerging metabolic genotypes were associated with higher AMR. Among NVTs 17F, 10A, 23B, 34 and 38 we show that MTs 109, 74, 93, 22 and 120 (respectively, Fig. 2) were more frequently identified in the later surveillance period (Fig. S3), indicating a shift in the dominant genotype observed within each serotype. In four of these NVTs (17F, 10A, 23B and 38), the emergence of MTs in later surveillance was associated with increased AMR: either in terms of an increased MIC for penicillin (genetically assessed, Fig. 3) or in terms of presence of other antibiotic resistance genes such as *tetM* and *ermB* (Fig. S4). In serotypes 17F, 23B and 38, there was an increase in the frequencies of MT114, MT97 and MT122, respectively. In each case, the MT that emerged showed a higher penicillin MIC, compared to MTs common in the bacterial population in the earlier surveys. Interestingly, the increased frequency of MT46 of serotype 34 in later surveillance surveys without exhibiting an increase in AMR suggests a variety of forces that drive the selection and restructuring of bacterial populations.

There were no significant changes (*p* > 0·05, Fisher’s exact test) in carriage frequency of the majority of the VTs over the duration of the surveillance period - i.e., they did not increase nor decrease when comparing the early and later periods of surveillance. These included, 3, 19F and 23F (Fig. 1 and S3). Serotypes 3 and 23F in particular were found at relatively high frequencies after vaccine introduction in other settings^37,38^. Contrary to what was described with NVTs, the persistent VTs in this dataset were characterised by the presence of a dominant MT (MT9 for serotype 3, MT3 for serotype 23F) consistently present during the survey period, showing increased penicillin MIC and the presence of antimicrobial resistance genes (Fig. S5).

Within those serotypes where we identified genotype switch events (namely, 17F, 10A, 23B, 38 and 23B), we investigated genetic relatedness amongst MTs and time-calibrated phylogeny, to reconstruct the emergence of those recent dominant MTs. We assessed the genetic relatedness between MTs, hence their likelihood to have recently expanded clonally, by calculating the core genome SNP (single nucleotide polymorphism) divergence between MTs (Fig. S6). For serotypes 38, 17F and 34, recently identified MTs were characterised by lower genotypic difference (lower SNP variance, serotype 38 MT120-28 *p* = 0·016, serotype 17F MT109-66 *p* < 0·0001, serotype 34 MT33-53 *p* < 0·0001, *F*-test for variances, Fig. S6), indicating that these isolates expanded clonally. This recent clonal expansion was confirmed by the temporal signal identified in the phylogeny (Fig. S7). Root-to-tip regression highlighted that the emergence of the recently identified lineages (MT109 and MT120) in serotype 17F and 38 was likely to have happened before the year 2011 (17F MT114: 2000·9 [95% CI 1988·35–2008·1] node 95, 2009·7 [2005·7–2012·3] node 94; for 38 MT122: 2009·3 [2000·3–2013·3] node 85, 2007·5 [1996·7–2012·2] node 84, 2004·1 [1985·2–2011·4] node 83). Thus, none of these MTs are likely to have originated after PCV13 introduction, but rather we propose that they existed as variants at low frequency before vaccine introduction that underwent clonal expansion. It was not possible to temporally calibrate the phylogeny of serotypes 23B, 10A and 34, hence we could not infer the time of emergence of those lineages. This may be due to the smaller sample size of some lineages (i.e., in serotype 10A) or to lineages that originated much earlier than their isolation in this study.

### Emerging metabolic genotypes show multiple adaptations

**Fig. 4:**
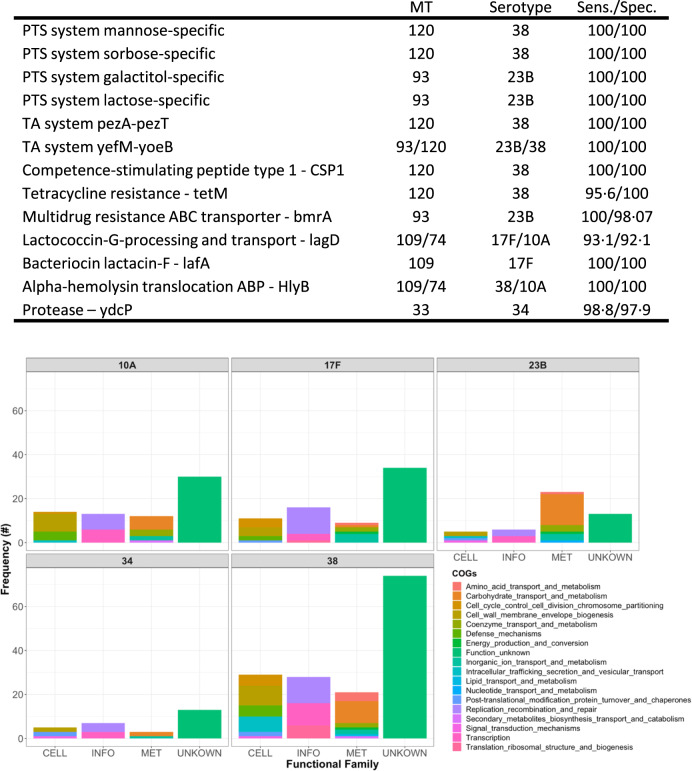
Pan-GWAS analysis results showing typical genes associated with expanding metabolic genotypes. The table shows examples of genes specific of MTs that expanded at late stages of carriage survey identified via pan-GWAS. Columns show the annotation of the genes or the gene system, the MTs and serotypes in which the gene was found, the sensitivity and specificity related to its presence in the MT reported in the table (and its absence in the other MTs). Full table is shown in supplementary material (Supplementary Data 1). All *p*-values (adjusted using a Bonferroni correction) were <0·0001, as reported by Scoary. The barplot graph shows clusters of orthologous genes enriched in Serotype 10A (MT74), 17F (MT109), 23B (MT93), 34 (MT33), and 38 (MT120). Each barplot shows the number of genes in each functional class for the dominant metabolic type in the 5 serotypes, as reported by the pan-GWAS analysis. The columns refer to the functional classes “Metabolism” (MET), “Cellular processes and signalling” (CELL), “Information storage and processing” (INFO), or poorly characterised genes (UNKNOWN).

Having demonstrated a shift in MTs in serotypes 10A, 17F, 23B, 34 and 38 associated with increased AMR during the post-vaccine introduction observation period, we then assessed whether these emerging MTs had associated virulence characteristics that could convey a competitive advantage. To do this, we investigated the accessory genome using a pangenome wide association study approach (pan-GWAS^39^). We identified 93, 73, 51, 30 and 175 genes present exclusively in the most common emerging metabolic profiles of serotypes 10A, 17F, 23B, 34 and 38, respectively (Fig. S8; full gene list is reported in Supplementary Data 1; examples of genes associated to each MT analysed are shown in Fig. 4). Functional annotation showed that these included genes responsible for metabolism and carbohydrate transport. In serotype 38 MT120, for instance, mannose- and sorbose-specific transporter genes, absent in other MTs, were identified. MT93 serotype 23B was characterised by lactose and galactiol transporters (Fig. 4) which were absent in other MTs. The emerging MTs were also characterised by the presence of bacterial defence systems, such as multidrug resistance ABC transporters, restriction enzymes and antimicrobial resistance genes (Fig. 4).

The MT93 and MT120 of serotypes 23B and 38 accessory genomes encode several Toxin-Antitoxin (TA) systems. Variants of the *pezAT* system was present in MT93 and MT120 and a *yefM*-*yoeB* system was present in MT120. This particular TA-system is located in a previously described integrative-conjugative region (ICE)^40^, which is absent from the non-MT120 strains. The ICE region is present in several non-pneumococcal *Streptococci*, such as *S*. *dysgalactiae* and *S. suis*^41^, suggesting that its acquisition may have occurred via horizontal gene transfer (HGT). This ICE also carries specific metal transporters, phage resistance and competence systems, as well as a previously described agglutinin receptor involved in colonisation^40^.

To assess the generalisability of our findings, we undertook a wider comparison using pneumococcal genomes sequences from other African countries^21,42^. This revealed the presence of analogous MTs outside Malawi. In particular, MT93 and MT120 (associated with serotypes 23B and 38 respectively) have been identified after vaccine introduction in invasive pneumococcal disease isolates in both South Africa and The Gambia^42^. Rather than originating from a common source, differences in the core genome SNPs among MT120 strains isolated in South Africa and in Malawi (~6000 SNPs, Fig. S9) suggest that these different lineages originate from different ancestors that have subsequently converged to the same MT.

Together, these data indicate that a range of potentially evolutionary relevant genes are associated with the emerging MTs (Fig. 4). In populations with a high force of infection and host vulnerabilities such as malnutrition and HIV, this potential for competitive advantage in nutrient transport, cellular metabolism, nasopharyngeal epithelial colonisation and AMR could lead to a resurgence in invasive NVT pneumococcal disease.

### Phenotypic characterisation of emerging MT reveals adaptations that may confer competitive advantage

To further explore this potential competitive advantage amongst emerging NVT metabolic genotypes with increased AMR, we compared MT shifts within serotype 38 and serotype 23B in several in vitro phenotypic assays. Rather than use a very large panel of strains that captures the variation within the accessory genome, we used representative strains of these MTs. To support this approach, we have assessed within MT genetic variation of virulence factors implicated in colonisation^43^ (Supplementary Data 4). We assessed amino acid sequence similarity rather than nucleotide similarity, because although nucleotide sequences would likely result in more variance, the impact of synonymous single nucleotide polymorphism on virulence is uncertain. To avoid missing potential changes in virulence, a conservative 95% amino acid similarity cutoff point was chosen. This analysis revealed that the variance in amino acid sequences of virulence genes ranged from 0 (540/865, 62.4%) to 2.54 (MT55, virulence gene pce/cbpE), with a mean variance of 0.03. Generally, we observed little variance in the amino acid sequences across virulent genes in metabolic types, with 10 out of 16 virulence genes having a mean average variance of less than 0.01. Together this indicates that there is little variance in the virulence gene repertoire of isolates belonging to the same MTs.

**Fig. 5:**
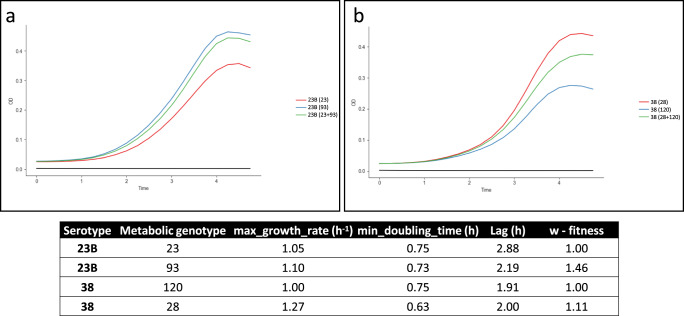
Growth curves and inferred in vitro fitness of different metabolic types in serotype 23B and serotype 38. The growth curves of the MTs comprising serotype 23B (**a**) and 38 (**b**) separately (blue and red curves) and in an in vitro co-culture (green curve). For each curve the MT or the combination of MTs is reported in parentheses. The table shows the growth parameters as predicted by Curveball (nu-deceleration parameter, q0-initial physiological state, r-specific growth rate in low density). These experiments were performed on at least three independent occasions with 16 replicates.

Our phenotypic experiments aimed at representing some of the challenges of bacterial lifestyle in the nasopharyngeal niche. Firstly, we cultured combinations of different MTs in standard growth medium. The growth parameters and the in vitro fitness were calculated by *Curveball*^44^ for MTs 28 and 120 for serotype 38 and MTs 23 and 93 for serotype 23B (Fig. 5). This software fits the growth curves of the different bacterial strains to several growth models and attempts to predict which one would have a competitive advantage over the other while sharing resources.

Within serotype 23B, the emergent strain MT93 reached a higher maximum optical density (OD) than MT23 (Fig. 5). Using the Baranyi-Roberts model to approximate co-culture growth, we found that MT93 is expected to have a higher in vitro fitness than MT24. Within serotype 38, the emergent strain MT120 grew at a lower rate and to a lower maximum OD than MT28 (Fig. 5) and had a lower predicted fitness than MT28. Given the opposing findings, we expanded the growth curve experiments to include dominant and non-dominant MTs within serotype 3 and 23F. We observed nearly identical growth patterns for serotype 23F strains, with a higher stationary-phase autolysis rate for serotype 23F’s dominant MT3 compared to the non-dominant MT123. Moreover, we observed a slightly higher growth rate for the non-dominant MT60 (ST5435) compared to the dominant MT9 (ST700) for serotype 3 strains (Fig. S10). Thus, while some emergent MTs have a growth advantage in vitro, others do not. However, all emergent and dominant MTs within serotype 23B, 38, 3 and 23F had a higher MIC to penicillin compared to their non-dominant counterparts (Figs. 3, S5).

**Fig. 6:**
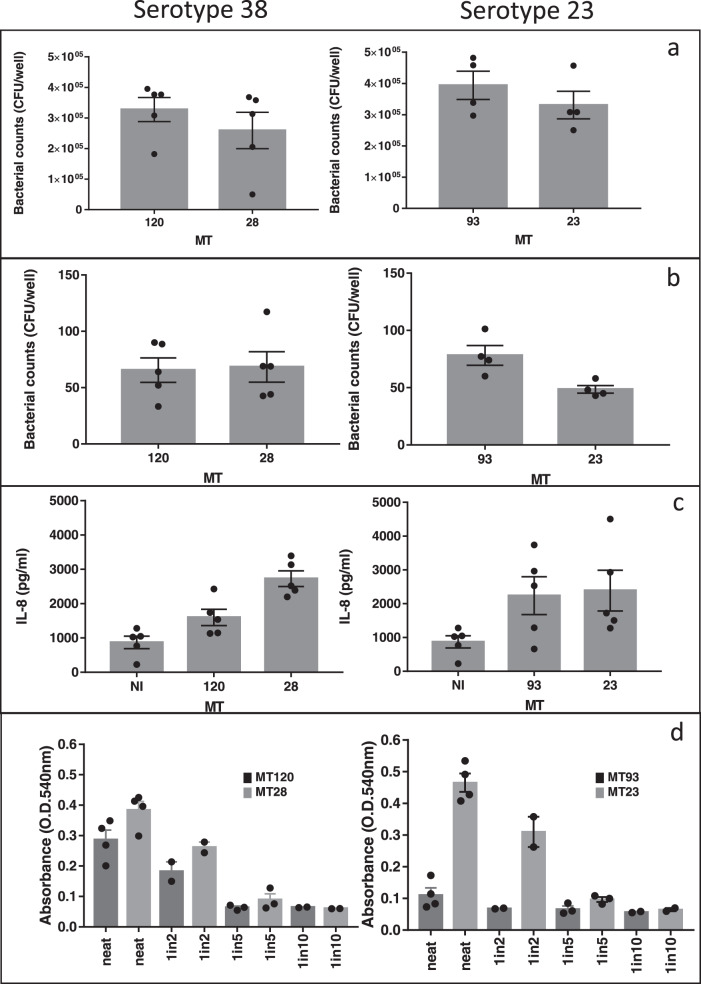
Association and invasion to epithelial cells, IL-8 secretion and haemolysis for MT120 and 28 (serotype 38) and MT93 and 23 (serotype 23B). **a** Number of bacteria associated with epithelial cells, recovered after 3 h of incubation, **b** Number of intracellular bacteria after 3 h incubation and 1 h with gentamicin treatment, **c** IL-8 produced by epithelial cells, incubated with bacterial cells for 6 h. NI non-infected control. **d** haemolysis promoted by the different bacterial strains (lower OD corresponds to higher haemolysis), for different bacterial dilutions (neat~1·5 × 10^6^ bacterial cells. Results for 1/2, 1/5 and 1/10 dilutions are shown). Representative strains from each MT were used in these experiments. Each experiment was repeated at least 3 times independently. In **a**, **b**, and **c**, dots on the bar plots show the result of each repetition. All *p*-values derived using Wilcoxon ranked-sum test: **a** serotypes 38 and 23, *p*-values = 0.31 and 0.34, respectively; **b** serotypes 38 and 23 *p*-values = 1 and 0.03, respectively; **c** NI vs MT120, *p*-value = 0.032; NI vs MT28, *p*-value = 0.16; MT28 vs MT120, *p*-value = 0.42; NI vs 93, *p*-value = 0.1; NI vs 23, *p*-value = 0.016; 93 vs 23, *p*-value = 1; **d** MT120 vs MT28: neat, *p*-value = 0.11; 1-in-2, *p*-value = 0.33; 1-in-5, *p*-value = 0.4; 1-in-10, *p*-value = 0.3; MT93 vs MT23: neat, *p*-value = 0.03; 1-in-2, *p*-value = 0.33; 1-in-5, *p*-value = 0.2; 1-in-10, *p*-value = 0.3. Data are represented as mean values and error bars are the standard errors of the mean. All *p*-values reported are for two-sided statistical tests.

The epithelial colonisation process is a prerequisite for both disease and onward transmission to another host^45^. We therefore then tested representative *S. pneumoniae* strains from these MT pairs in a well-standardised Detroit 562 human nasopharyngeal epithelial infection model^46^. Within serotype 38, we observed no difference in the ability to colonise the epithelial cells between MTs 28 and 120 (Fig. 6a). In contrast, serotype 23B, MT93 was internalised into epithelial cells at a higher frequency than MT23 (Fig. 6a, b) even though both genotypes were characterised by the same ability to associate with epithelial cells. We have previously suggested that differences in this so-called epithelial “microinvasion” may be fundamental to the outcome of colonisation, explaining strain differences in the potential of *S. pneumoniae* to cause invasive disease, persist during carriage, and be transmitted from person-to-person^46^.

Other phenotypic characteristics in different MTs were assessed, such as their ability to stimulate the production of the phagocyte chemoattractant IL-8 in epithelial cells (Fig. 6c) and their haemolytic potential, a measure of functional pneumolysin production (Fig. 6d). Serotype 38’s MT120 stimulated lower IL-8 production, compared to MT28, while the two MTs in serotype 23B did not stimulate a higher IL-8 production when compared to the non-infected control (Fig. 6c). The haemolytic potential of serotype 23B’s MT23 and serotype 3’s MT9 were higher than their serotype matched counterparts, while no significant difference was identified between serotype 38 and 23F strains (Figs. 6d, S10).

**Fig. 7:**
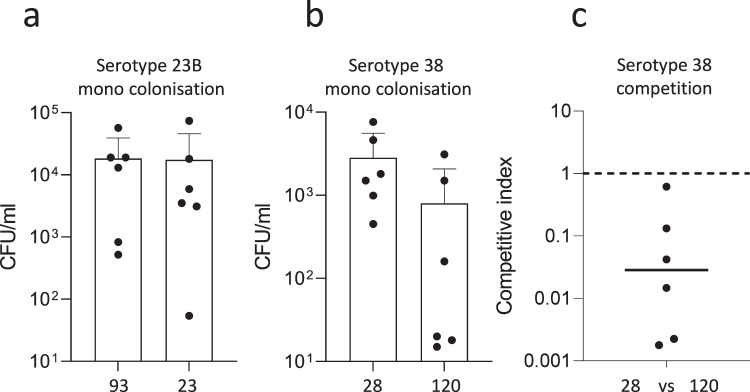
Murine colonisation density for MT93 and MT23 (serotype 23B) and MT120 and MT28 (serotype 38). CFUs recovered from nasopharyngeal washes of CD1 outbred mice inoculated intranasally singly with serotype 23B MT93 or MT23 (**a**), singly with serotype 38 MT120 or MT28 (**b**). In **c** CFUs from a competition experiment with a 1:1 ratio of serotype 38 MT120 and MT28, seven days post inoculation, are transformed into the competitive index. Six mice were used for each condition. All *p*-values derived using Wilcoxon ranked-sum test: **a** *p*-value = 0.7. **b** MTs 120 and 28 *p*-value = 0.06; **c** MTs 93 and 23 *p*-value < 0.001. All *p*-values reported are for two-sided statistical tests.

Finally, we employed a 7-day murine colonisation model to investigate the ability of the MTs to colonise the nasopharynx in competition^47^. Despite similar levels of epithelial adherence and internalisation in vitro, serotype 38 MT28 had a tendency to colonise the murine nasopharynx to a greater extent than the emerging genotype MT120, when inoculated singly (Wilcoxon, *p* = 0.06), and had a significant advantage in a competition (Wilcoxon, *p*-value < 0.001, Fig. 7b, c). In contrast, there were no differences in the number of CFUs recovered from the murine nasopharynx for serotype 23B MT93 and MT23 (Fig. 7a).

Together these data suggest that the emerging MTs have not arrived at a single adaptive profile but have undergone a variety of changes (growth; epithelial invasion; inflammation; and pneumolysin production) that may have enabled them to become more prominent in the context of vaccine introduction. However, while these experiments provide proof of concept for our approach to the phenotypic characterisation of emerging MTs in a vaccine-exposed population, further validation is needed through similar analyses in other populations.

## Discussion

How a pneumococcal population evolves both under natural or vaccine-induced pressures remains an open question. It has been long known that *S. pneumoniae* is characterised by a high level of genome plasticity, and this species diverges more quickly than many other mucosal bacterial species associated with vaccine preventable disease^25,48^. Here, using a large post-PCV introduction pneumococcal carriage surveillance dataset from an urban population in Malawi, we describe the emergence of pneumococcal lineages with unique virulence, AMR and metabolic characteristics. Rather than using the populational structural methods of the Pneumococcal Molecular Epidemiology Network (PMEN)^49^ or the Global Pneumococcal Sequencing (GPS) consortium^42^, our genotyping approach defined pneumococcal lineages based on metabolic genotypes (MT) that we have proposed^27^ may give a wider understanding of the biological drivers to changes in pneumococcal population structure.

We show that in addition to serotype replacement, there has been a shift in the profile of AMR MTs among NVTs characterised by exclusive genes responsible for metabolism and carbohydrate transport, and toxin-antitoxin systems located in an integrative-conjugative region suggestive of horizontal gene transfer. Phenotypically, representative strains from these emergent genotypes were found to have differential growth, haemolytic, inflammatory or epithelial association/ invasion traits that may confer advantage in the nasopharyngeal niche. However, the genotype-phenotype relationship is multidimensional. For example, our finding that while similar single strain colonisation levels were achieved in a murine model of pneumococcal carriage, when inoculated together, serotype 38’s MT28 outcompeted the emerging MT120 genotype is contrary what might be anticipated for an emerging genotype. We maintain that the emergence of such genotypes results from multiple phenotypic adaptations that may impact on both colonisation efficiency and transmission. Indeed, together these findings support theoretical models that predict that PCV introduction disrupts complex competitive interactions between colonising pneumococci, with the potential to promote replacement with fitter, more virulent, and more antimicrobial resistant existing pneumococcal lineages^22,27^. The serotypes contained within PCVs are selected primarily on the basis of invasive potential in infants^21^. We postulate that if these emergent AMR NVT MTs become fixed in the population, out-competing NVTs with less invasive/ disease-causing potential, they may lead to lower vaccine effectiveness against pneumococcal disease overall. We also observed stable MTs among VTs and although our observations are limited to the post-PCV13 era, we speculate that these genotypes may confer a competitive advantage leading to inadequate PCV13 effectiveness in the control of VT carriage, including serotypes 3, 19F and 23F. While the increased penicillin MICs that were observed amongst emerging or persistent MTs do not cross the thresholds that would likely lead to treatment failure for diseases such as pneumonia or meningitis, in this heavily antibiotic exposed population^50^, we speculate that if this upward drift in MIC continues, and it may confer a competitive advantage in the nasopharyngeal niche.

The overall effect of the shift in MTs that we have observed is dependent on their colonisation potential, transmissibility and their relative invasiveness^21^. These MTs have emerged within serotypes that are known to be important contributors to IPD and are commonly carried. Serotype 38 has high heterogeneity in its invasive potential in South Africa and the USA;^21^ serotype 23F ST2059/MT3 was reported to have a higher risk for invasiveness in strains isolated in South Africa and in the USA; serotype 23B ST4423/MT93 was identified in IPD in South Africa and Israel; and serotype 35B, which also expanded in frequency in this study, was earlier highlighted as an expansion serotype in Malawi IPD^21,42^. Moreover, serotypes 23B and 38 are among the top 10% of serotypes causing IPD after vaccine introduction in Europe, Asia and US^51^.

The emergence and expansion of particular NVTs in the period after PCV introduction has been widely reported^21^. The serotype switching processes and dynamics highlighted here have also been reported by others^23,42^. For instance, the serotype switch event involving ST8672 (serotypes 3 and 7F) was also identified in a post-vaccination carriage study in northern Malawi^23^. Similarly, the Global Pneumococcal Sequencing study, identified ST10599 and ST989 as part of switch events, highlighting how these mechanisms are common in pneumococcal genotype and serotype dynamics^21^. Our finding of a shift in MTs in Malawi appears to have also been seen in other African countries, leading us to hypothesise that these MTs have become more prevalent across the continent following PCV introduction.

A theme that has emerged from our analysis is that although the emergent MTs have successful metabolic, virulence and AMR properties that may confer a competitive advantage over other MTs, we did not identify genetic or phenotypic traits that suggest a single adaptive profile amongst the emergent MTs. In contrast, we identified a complex set of multiple different adaptations that have the potential to provide an advantage in distinct ecological niches. For instance, some MTs are characterised by sugar transporters, TA-systems and bacteriocins production systems, which would confer competitive advantage while sharing resources. Carbohydrate metabolism is an increasingly recognised determinant of pneumococcal niche adaption and progression to disease^52^. In addition, a characteristic of the MTs that appeared to have emerged in recent years in our study population is the presence of genes that encode particular toxin/antitoxin systems. These systems are present in a variety of bacterial species both at a plasmid and chromosomal level, and involved in a variety of cellular processes–including virulence, environmental stress response and niche adaptation^53,54^. The pan-GWAS analysis highlights the presence of virulence factors in expanding MTs: in MT33 for instance the presence of a *ydcP* protease, a collagenase which facilitates breaking of extracellular structures and a known virulence factor in other bacterial species^55^, identified to be associated with pneumococcal IPD;^56^ and in MT109 and MT74 of a haemolysin (pneumolysin) translocation ATP-binding protein gene, known virulence factors for *S. pneumoniae*. Together, these observations indicate that the expanding metabolic types accumulate characteristics that may affect niche competition and virulence.

Our phenotypic analysis of representative strains further highlights the complex and sometimes conflicting effects of the genotypic shifts identified. Recent studies have described bacterial fitness in different terms, adapting its definition to the available data. For instance, the description of negative frequency dependent selection in *S. pneumoniae* populations, whereby bacterial fitness is affected by the frequency of rare accessory genes^57,58^. We have used growth curve analysis to infer relative, mixed-culture bacterial fitness^44^. In combination with previous modelling analyses, the results described in this work are consistent with a complex and diverse behaviour of a *S. pneumoniae* population under vaccine pressure. Serotype 23B MTs for instance, behaves similarly to the VIMS model description whereby the fitter genotype which was suppressed beforehand expands after vaccine introduction due to niche clearance (Fig. 3). On the other hand, serotype 38’s MTs behave in accordance with the hypothesis of an increase in frequency of AMR NVTs, driven by vaccination^22^. An antibiotic susceptible MT of serotype 38 exhibited a higher intrinsic fitness, as determined by the growth curve analysis. However, we postulate that this MT was overtaken by another, antibiotic resistant MT of serotype 38, when vaccination alleviated the competition pressure from VTs.

We also found that the most common recent strain of serotype 23B, namely MT93, is less haemolytic (pneumolysin mediated) than MT23 and more successful in invading nasopharyngeal cells in vitro but has similar colonisation levels in a murine colonisation model. On the other hand, for serotype 38, the recently dominant MT120 stimulated less IL-8 production but was less successful in the murine colonisation model, when compared to the less common metabolic genotype MT28. Pro-inflammatory interleukin-8 attracts neutrophils to the site of infection^59^ and therefore by evading the immune system MT120 may have a competitive advantage. While the similar or lower murine colonisation densities seen with the emerging genotypes may seem disadvantageous, we propose that features such as AMR, together with measured and unmeasured virulence traits, may outweigh these effects during human carriage.

Importantly, our approach to metabolic genotyping should not be viewed as a proposed alternative to established pneumococcal strain-typing systems. Instead, the genetic and phenotypic analyses, and their varying results, highlight that pneumococcal evasion of the immune system, or more successful colonisation of different ecological niches, is both complex and multifactorial. This is often the case in evolutionary landscapes^60^, where a simple explanation of complex phenomena is unattainable. We have not identified a common phenotype characteristic between all the recently expanded genotypes, but we highlighted several differences that could help the success of each genotype in different environments and at different time points^27^. However, although a very large undertaking requiring analyses from different populations under vaccine pressure, if phenotypic characteristics of the multiple genotypes that have emerged since PCV13 introduction are characterised across both in vitro and in vivo platforms, common profiles may emerge.

Our study has several limitations. First, the lack of genomic data prior to the introduction of PCV in Blantyre. Pneumococcal carriage surveys have been undertaken in the pre-PCV era in Malawi but not with the same systematic approach, and largely not in the same populations geographically or in people living with HIV^31,61–64^. To avoid these biases, we have therefore not undertaken a direct comparative analysis. However, we have been able to leverage this large dataset to capture ongoing changes in a population with high vaccine coverage^16,29^. Because of the cross-sectional nature of the surveillance contributing to the Blantyre collection we were not able to track the emergence of MTs within individual hosts over time. Therefore, as is common in observational studies, we have not been able to attribute causality to routine vaccine introduction.

Second, the MTs identified in this study in Malawi may not be fully generalisable to other pneumococcal populations. For example, the core genome underlying our analysis is likely to change with varying number of isolates and genetic heterogeneity. Hence, population-specific analyses will be required to address particular hypotheses in other populations.

Third, our experimental observations are limited by the use of a single representative strain from each MT and although we saw little variation in virulence genes within MTs, there is the potential that our model systems may not fully capture the competitive advantage of emerging MTs. Rather than analysing the large number of strains required to capture the full extent of the genetic variation within the accessory genome, to further validate our findings, we advocate for similar analyses in other populations under vaccine pressure. We used an endpoint-outcome of murine colonisation to assess differences in carriage potential. However, a kinetic model, as described by Zafar et al.^65^, may have provide greater insights into competitive advantage. Nonetheless, our experiments have highlighted the complex, multidimensional nature of pneumococcal dynamics. Comprehensive future work will be needed to pinpoint the effects of each dimension of the fitness landscapes of different populations.

In conclusion, we postulate that in this high burden vaccine-exposed population, AMR and a range of genotypic and phenotypic traits may facilitate the expansion of MTs in the community. Vaccine strategies that increase PCV valency may be insufficient to interrupt this process^66^. The multiple adaptations by these MTs extend beyond simple serotype replacement, and successful adaptation could further undermine vaccine control and promote the spread of AMR.

## Methods

### Bacterial isolates and carriage surveys

The strains analysed in this study were isolated as part of a prospective observational study using stratified random sampling to assess pneumococcal nasopharyngeal carriage in Blantyre, Malawi^16^. In brief, sampling consisted of twice-annual rolling cross-sectional surveys over the course of 4 years amongst PCV13-vaccinated children 2–7 years old, PCV13-unvaccinated children 5–10 years old, and HIV-infected adults 18–40 years old on ART. Nasopharyngeal sample collection, storage and testing were all performed using standard approaches^16^. As commonly done for pneumococcal carriage surveys, one colony was selected for purification and subsequent serotyping from each sample, selected by its ease of sampling and minimisation of contamination risk. 2804 carriage isolates were thus selected at random from the 8 surveys to develop the WGS libraries. Isolates from the HIV-infected adults were sequenced from surveys 1, 2, 5 and 6 only. Particularly in children, multiple serotype carriage as determined by DNA microarray, is relatively high in this population^31,32^. However, our comparative analysis shows good concordance between a single *S. pneumoniae* colony selected for phenotypic serotyping and the dominant serotype determined by microarray molecular serotyping^32^.

### DNA sequencing and assembly

NP swabs were stored in skim-milk-tryptone-glucose-glycerol (STGG) at −80 °C. After being thawed and vortexed, 30 µl of STGG was plated on gentamicin-sheep blood agar (SBG; 7% sheep blood agar, 5 µl gentamicin/mL) and incubated overnight at 37 °C in 5% CO2. Plates showing no *S. pneumoniae* growth were incubated overnight a second time before being reported as negative. *S. pneumoniae* was identified by colony morphology and optochin disc (Oxoid, Basingstoke, UK) susceptibility. The bile solubility test was used on isolates with no or intermediate optochin susceptibility (zone diameter < 14 mm). A single colony of confirmed pneumococcus was selected and grown on a new SBG plate as before. Growth from this second plate was used for serotyping by latex agglutination (ImmuLex™ 7-10-13-valent Pneumotest; Statens Serum Institute, Denmark)^16,32^. DNA was extracted from an overnight lawn plate culture from isolates archived after serotyping, using DNAeasy blood and tissue kit (Qiagen) following the manufacturer’s guidelines and sequenced using HiSeq4000 (paired-end library 2 × 150) platform at Oxford Genomics Centre UK.

Raw DNA reads were trimmed of low-quality ends and cleaned of adaptors using Trimmomatic software (ver. 0·32)^67^. De novo assembly was performed with SPAdes software (ver 3·8·0)^68^, using a sample of 1,400,000 reads and k-mer values of 21, 33, 55, and 77. De novo assemblies were checked for plausible length (between 1,900,000 and 2,200,000 bp), annotated using Prokka (ver. 1·12)^69^, and checked for low-level contamination using Kraken (ver. 0·10·5)^70^ and the module “rMLST species id” on the pubmlst database (pubmlst.org)^71^. In cases for which more than 5% of the contigs belonged to a species different from *Streptococcus pneumoniae*, the genome sequence was not included in any further analysis. Resulting assemblies are available on pubmlst.org (https://pubmlst.org/organisms/streptococcus-pneumoniae). Metadata of the strains used in this work are reported in Supplementary Data 2.

### Definition of serotype, multilocus sequence type, global pneumococcal sequencing cluster and AMR

Serotypes were determined via DNA sequence, using the PneumoCaT software (Ver. 1·2·1, https://github.com/phe-bioinformatics/PneumoCaT)^72^. The tool reports serogroup 15 as serotype 15 A, 15B, 15 C and 15B/C. Phenotypic serotype definition by latex agglutination was used as validation of the genetic serotype definition, observing a concordance of over 90%, in alignment with previous estimations^32^. For the analyses in this work, serotype determined by DNA sequencing was used.

Multilocus sequence types (MLST, STs) were assigned using the databases from the allelic profiles of 7 housekeeping genes (*adhP, pheS, atr, glnA, sdhA, glcK*, and *tkt*). BLASTn (ver. 2·10·0 + ) was used to align the DNA assemblies to the DNA fragments typical of each allele for each housekeeping gene (parameters: *E*-value 1e − 10, minimum 95% identity, minimum 90% query coverage). This grouped strains into 200 unique STs. Strains which did not show a full set of housekeeping gene alleles or were not assigned to any previously described ST (*n* = 783) were double-checked for sequence contamination and assigned to a new sequence type definition. The database of MLST housekeeping fragments was downloaded from pubmlst.com/Spneumoniae in October 2019. The GPSC genetic typing method (Global Pneumococcal Sequence Clusters) was described in several recent publications^21,23,42^. We collected the dataset to assign the GPSCs from https://www.pneumogen.net/gps/assigningGPSCs.html and used PopPUNK (ver. 2·0·2)^73^ to cluster the isolates in the pre-defined GPSCs as described in Gladstone and colleagues in 2019^42^. AMR gene presence (for genes *cat, tetM, ermB, and mefA*) was detected via nucleotide-BLAST (*E*-value < 0.001, sequence coverage and identity >80%)^74^. For penicillin resistance, MIC was genetically assessed using the *pbp* genes allelic profile^75^.

### Pangenome, definition of metabolic profiles and phylogenetic analyses

In order to capture the total variability of the population, a pangenome was generated from the combined isolates from surveys 1 to 8 using Roary (ver. 3·8·0)^76^. Parameters for each run were: 90% of minimum BLASTp identity; MLC inflation value 1.5; with 99% of strains in which a gene must be present to be considered “core”. Saturation of the pangenome was assessed. The core genome of this bacterial population included 1061 genes which was consistent with previous estimates from Malawi^77^. The metabolic genes were selected via functional annotation of the core genome (*n* = 386 core metabolic genes)^27^ through the eggNOG database (http://eggnogdb.embl.de). This was done according to the KEGG Orthology (KO) groupings of the KEGG database^27^. Functional classes selected to define the core metabolic genome were: D-Energy production and conversion, E-Amino acid transport and metabolism, F-Nucleotide transport and metabolism, G-Carbohydrate transport and metabolism, H-Coenzyme transport and metabolism, I-Lipid transport and metabolism, P-Inorganic ion transport and metabolism, Q-Secondary metabolites biosynthesis, transport, and catabolism.

Metabolic profiles were defined using the following pipeline: (i) for each genome, the allelic profile of the metabolic core genes was defined using the Genome Comparator module of Bigsdb (bigsdb.com);^71^ (ii) the hamming distance between each allelic profile was calculated (*hamming. dist* function of base R 3·6, https://www.R-project.org/); (iii) the hamming distance between each isolate was used to hierarchically cluster the population (*hclust*); (iv) the hierarchical clustering dendrogram was cut at midpoint to define the discrete metabolic profiles.

Maximum-Likelihood, recombination censored phylogeny was calculated with ClonalframeML (ver. 1·12)^78^, under a generalised time-reversible model with 100 bootstrap replicates. Core genomes used to produce ML phylogeny were calculated separately for the isolates belonging to the serotypes of interest (17 F, 10 A, 38, 34 and 23B) with Roary as described above. SNPsites (Ver 1·04)^76^ was used to identify the variable part of the core genome alignment, which was then used for phylogeny reconstruction. Root-to-tip regression and phylogeny dating were calculated with the BactDating R package (https://github.com/xavierdidelot/BactDating, 700000 permutations)^79^.

The pan-GWAS analyses were carried out using Scoary (https://github.com/AdmiralenOla/Scoary)^80^ as described by Gori and colleagues in 2020^39^.

To ascertain the expected properties of the metabolic genes, an analysis of linkage disequilibrium (LD) using the approach taken by Watkins et al.^27^. The analysis used the ATCC 700669 reference genome and 100 randomly selected loci pairs to compare LD D’ values in metabolic and non-metabolic genes, resulting in an average D’ values of 0.93 and 0.9, respectively (Fig. S11). We note that our larger dataset forces the core genome to be more conserved, leading to higher D’ values than in Watkins et al.

### Co-culture fitness and epithelial association/ invasion experiments

Strains BVY5TE, BVY11B (serotype 23B, MT23 and MT93 respectively), BVY2DJ and BVY123 (serotype 38, MT120 and MT28 respectively) were selected for phenotypic evaluation (Supplementary Data 2).

In vitro broth culture was carried out in order to apply the pipeline described by Ram and colleagues in 2019^44^. Isolates belonging to different MTs were harvested from a THY-10% glycerol stock and grown overnight on a Columbia agar plate with 5% horse blood (CBA) plate. The plate was used to seed 10 ml of Todd-Hewitt broth with 2·5% yeast extract (THY) to an optical density at 600 nm (OD_600_) of 0·5–1, which was then diluted to an OD_600_ 0·05 and used directly as starter in at least 16 replicate 200ul wells of a 96-well plate. Each experiment also contained a mixture of 100 μl each of the two strains. Each plate was incubated statically for 18–20 h, at 37 °C, with 5% carbon dioxide in a plate reader (Tecan Spark M20). OD_600_ was measured every 15 min. In order to explore the possibility that ill-defined components of THY may bias the differences seen, as well as to subject the cells to an environment more similar to the one found in the nasopharynx, chemically defined minimal medium proposed by Aprianto and colleagues^81^ was tested, but the isolates failed to grow in this medium.

Curveball (ver. 0·2·5) was used to fit each growth curve to a bacterial growth model and calculate each strain-relative fitness by modelling the behaviour of each strain in co-culture. As curveball was designed to calculate fitness in *E. coli* growth experiments, the growth curves were censored after each strain reached the maximum OD_600_, to correct for the characteristic *S. pneumoniae* autolysis.

Association and invasion experiments on human respiratory tract epithelial cells (Detroit 562) were carried out as reported by Weight and colleagues in 2019^46^. Briefly, human pharyngeal carcinoma Detroit 562 epithelial cells (ATCC_CCL-138) were grown in 10% FCS in alpha MEM media (Gibco). Confluent Detroit 562 (typically day 8 post-plating) were co-cultured with S*. pneumoniae* for 3 h in 1% FCS alpha MEM (MOI~1 cell:10 pneumococci). The medium was removed, and cells washed three times in Hanks Buffer Saline Solution (HBSS +/+, with calcium and magnesium, Gibco). Cells were incubated in 1% saponin for 10 min at 37 °C and lysed by repetitive pipetting. Dilutions of bacteria were plated on blood agar and colonies counted after 16 h. To quantify internalised bacteria, 200 µg/ml gentamicin was added for 1 h to the cells, which were washed another three times, before incubating with 1% Saponin and plating on blood agar plates. CFUs were counted after 16 h incubation at 37 °C, 5% CO_2_. For analysis of IL-8 secretion, Detroit 562 cells were infected with *S. pneumoniae* for 6 h and supernatant was collected. The protocol for the DuoSet® ELISA kit (R&D Systems) was followed according to manufacturers’ instructions.

Finally, for testing the haemolytic activity, bacteria were suspended in phenol free RPMI (Invitrogen) and incubated with 2% red blood cells (EO labs) in a U-bottom 96 well plate for 30 min. at 37 °C / 5% CO_2_. Saponin was used as a positive control for cell lysis. The plate was centrifuged at 1500 *g* for three minutes and supernatant was transferred to a new plate to read absorbance at 540 nm. All the abovementioned experiments were performed on at least three independent occasions.

### Variance between MTs

To determine the variance between isolates of the same MT virulence genes’ amino acid sequences, all protein-coding genes were first identified and translated into amino acid sequences from their assembled WGS using Prodigal^82^. The amino acid sequences were then compared to VFDB (Virulence Factor Database) amino acid sequences of known Pneumococcal virulent genes using BLASTP (particularly those involved in colonisation^43^), filtering out any hits that do not have a full alignment with the reference sequence, have gaps in the alignment, or have less than 95% sequence identity to that of the reference virulence genes’ amino acid sequence^83^.

### Murine colonisation experiments

Five-week-old outbred female CD1 mice (Charles River Laboratories) were inoculated intranasally with 5 × 10^6^ CFU *S. pneumoniae* under anaesthetic (isoflurane) for colonisation experiments. For competition experiments, 1:1 mix of serotype 38 MT28 and MT120 was used. Six mice were used for each experiment. Nasopharyngeal washes were obtained 7 days post inoculation and CFU enumeration performed using Columbia blood agar (CBA) plates supplemented with 4 µg/ml gentamicin. For competition experiments, plates were additionally supplemented with 10 µg/ml tetracycline, and the competitive index was calculated^84^.

All animal procedures were approved by the local ethical review process and conducted in accordance with the relevant UK Home Office-approved project license (PPL70/6510). Mice were housed for at least one week under standard conditions before use. Randomisation or blinding was not performed for these experiments.

Throughout the manuscript and supporting material, box plots represent the lower and upper quartiles as the box edges, a horizontal line represents the median, and whiskers are the minimum between 1.5-fold the interquartile range and the farthest observation from the quartiles.

### Reporting summary

Further information on research design is available in the Nature Portfolio Reporting Summary linked to this article.

### Supplementary information

Supplementary Information

Description of Additional Supplementary Files

Supplementary Data 1

Supplementary Data 2

Supplementary Data 3

Supplementary Data 4

Reporting Summary

## Data Availability

All required data are found in the Supplementary Material or archived online, as described in the Methods. All isolate sequences are available from NCBI (https://www.ncbi.nlm.nih.gov/bioproject/PRJNA1011974).

## Code availability

All data analysis protocols and software used are explicitly detailed in the manuscript and the supplementary files.
