# Supplementary material for: The metabolic, virulence and antimicrobial resistance profiles of colonising Streptococcus pneumoniae shift after PCV13 introduction in urban Malawi
Source: Nat Commun. 2023 Nov 17;14:7477. doi: 10.1038/s41467-023-43160-y (PMC10656543; doi:10.1038/s41467-023-43160-y)

Supplementary Material

Table S1 – Capsular switching events

For each ST we present the most common serotype (major), the least common serotypes (minor serotype, described in the text as switch serotypes) and whether each serotype is included in PCV13 (VT and NVT). Serotypes are based on PneumCat.

ST	Most common serotype		Less common serotype	PCV13 VT/ NVT	ST	Most common serotype		Less common serotype	PCV13 VT/ NVT
172	15B	NVT	15C	NVT	8672	3	VT	7F	NVT
			19F	VT	9523	18C	VT	18A	NVT
			15A	NVT				18F	NVT
			15BC	NVT	9552	20	NVT	3	VT
989	12F	NVT	19F	VT	10554	15A	NVT	11A	NVT
2059	23F	VT	13	NVT				19F	VT
3214	35A	NVT	11A	NVT				22A	NVT
			3	VT				9V	VT
			18A	NVT	10568	11A	NVT	3	VT
			18C	VT				11D	NVT
			35C	NVT	10587	7B_7C_40	NVT	19F	VT
4423	23B	NVT	23F	VT				19A	VT
5266	18C	VT	18B	NVT	10599	35B	NVT	14	VT
			22A	NVT				19A	VT
5435	3	VT	20	NVT				19F	VT
6279	23F	VT	23B	NVT	10603	15B	NVT	15BC	NVT
6441	48	NVT	3	VT				15C	NVT
7105	7B_7C_40	NVT	19A	VT				19F	VT
			19F	VT				15A	NVT
			3	VT	10660	6B	VT	6E	NVT
7653	6C	NVT	6D	NVT	11709	6B	VT	6E	NVT
			6A	VT	11770	16F	NVT	3	VT

Table S2 – Analysis of VT/nVT trends in different GPSCs.

Results from logistic regression models with VT and nVT as the dependent variable (coded as 1 and 0, respectively), and survey number as the independent variable, for each GPSC of those presented in Figure S2. p-values are not adjusted for multiple comparisons.

GPSC	Slope	p-value
5	0.188	0.194
9	-0.015	0.916
10	0.237	0.508
14	-0.063	0.743
22	0.012	0.911
26	-0.306	0.424
30	-17.063	0.997
40	-0.26	0.141
43	-0.255	0.169

92	-0.082	0.562
102	-0.164	0.703
116	-0.097	0.743
161	0.688	0.367
163	-0.152	0.644
184	0.122	0.673
228	-0.205	0.597
455	0.644	0.024

Table S3 – Analysis of VT/nVT trends in different STs.

Results from logistic regression models with VT and nVT as the dependent variable (coded as 1 and 0, respectively), and survey number as the independent variable, for each ST of those presented in Figure S2. p-values are not adjusted for multiple comparisons.

ST	Slope	p-value
172	0.306	0.432
989	-0.431	0.348
2059	0.064	0.834
3214	-0.304	0.205
4423	-0.942	0.14
5266	-0.356	0.199
5435	-0.049	0.925
7105	-0.207	0.513
9552	0.164	0.556
10554	-0.294	0.4
10568	-0.175	0.602
10587	0.021	0.921
10599	-0.109	0.712
10603	1.249	0.071

Figure S1 – frequency of predicted Serotypes (a), STs (b), and GPSCs (c) in the three cohorts (vaccinated, unvaccinated children and adults).

The frequency of genotypes and serotypes present in at least 10 strains is shown, non-typeable strains are excluded from plot (a) and (b). The proportions shown in each plot are relative to the separate cohort.

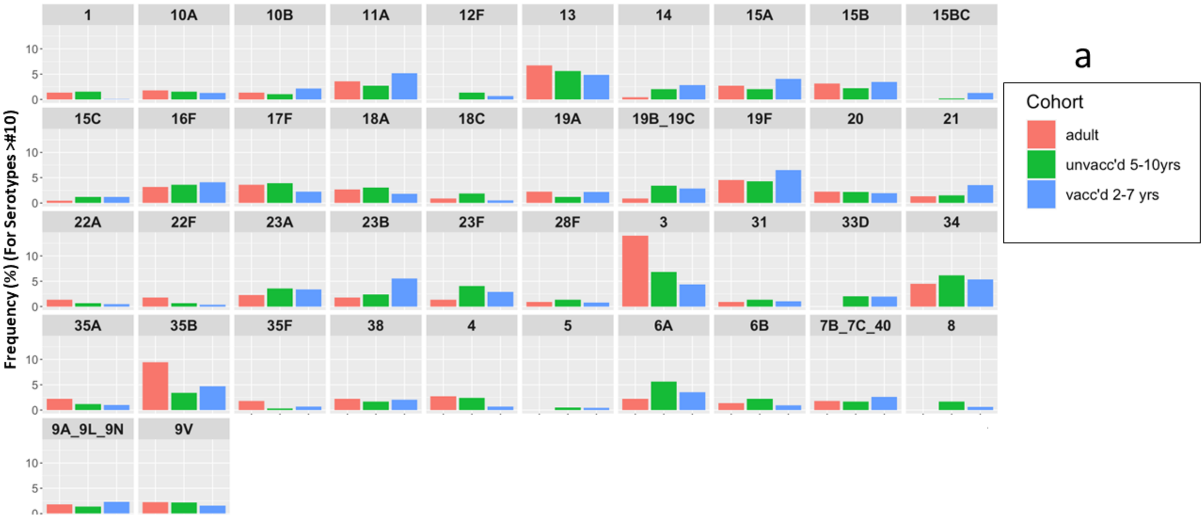

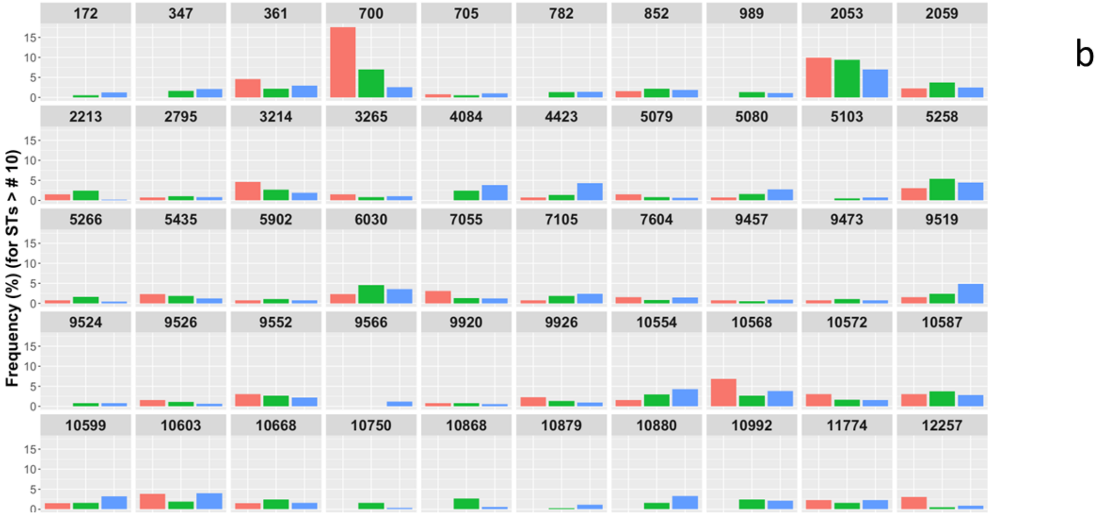

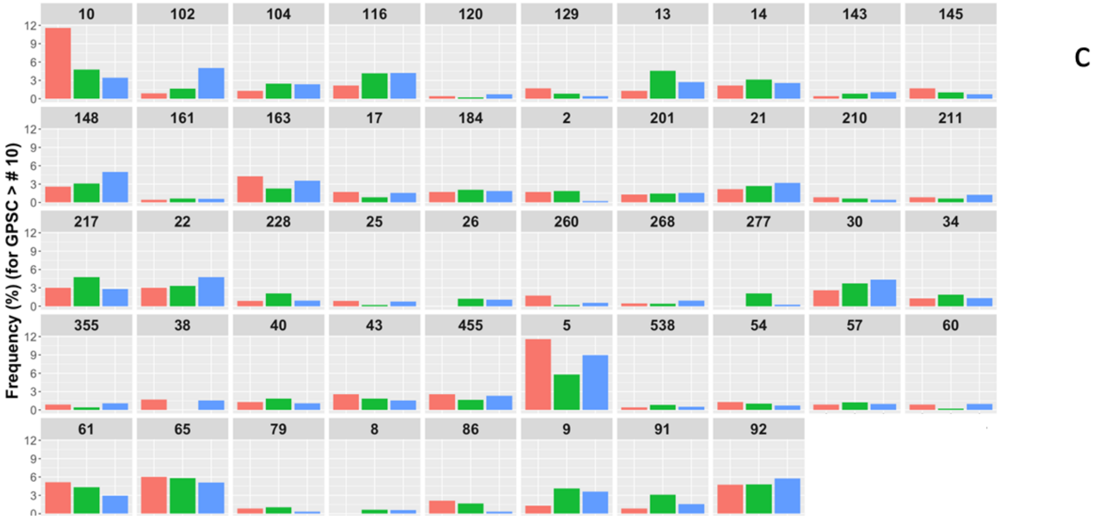
Frequency (%) (for GPSC > # 10)
10
102
104
116
120
129
13
14
143
145
148
161
163
17
184
2
201
21
210
211
217
22
228
25
26
260
268
277
30
34
355
38
40
43
455
5
538
54
57
60
61
65
79
8
86
9
91
92
c

Figure S2 – Number of strains identified as a vaccine- or non-vaccine serotype, in common STs (a) or GPSC (b) in time.

STs or GPSCs present in more than 10 isolates are shown. Non-typeable serotype strains are excluded from this analysis.

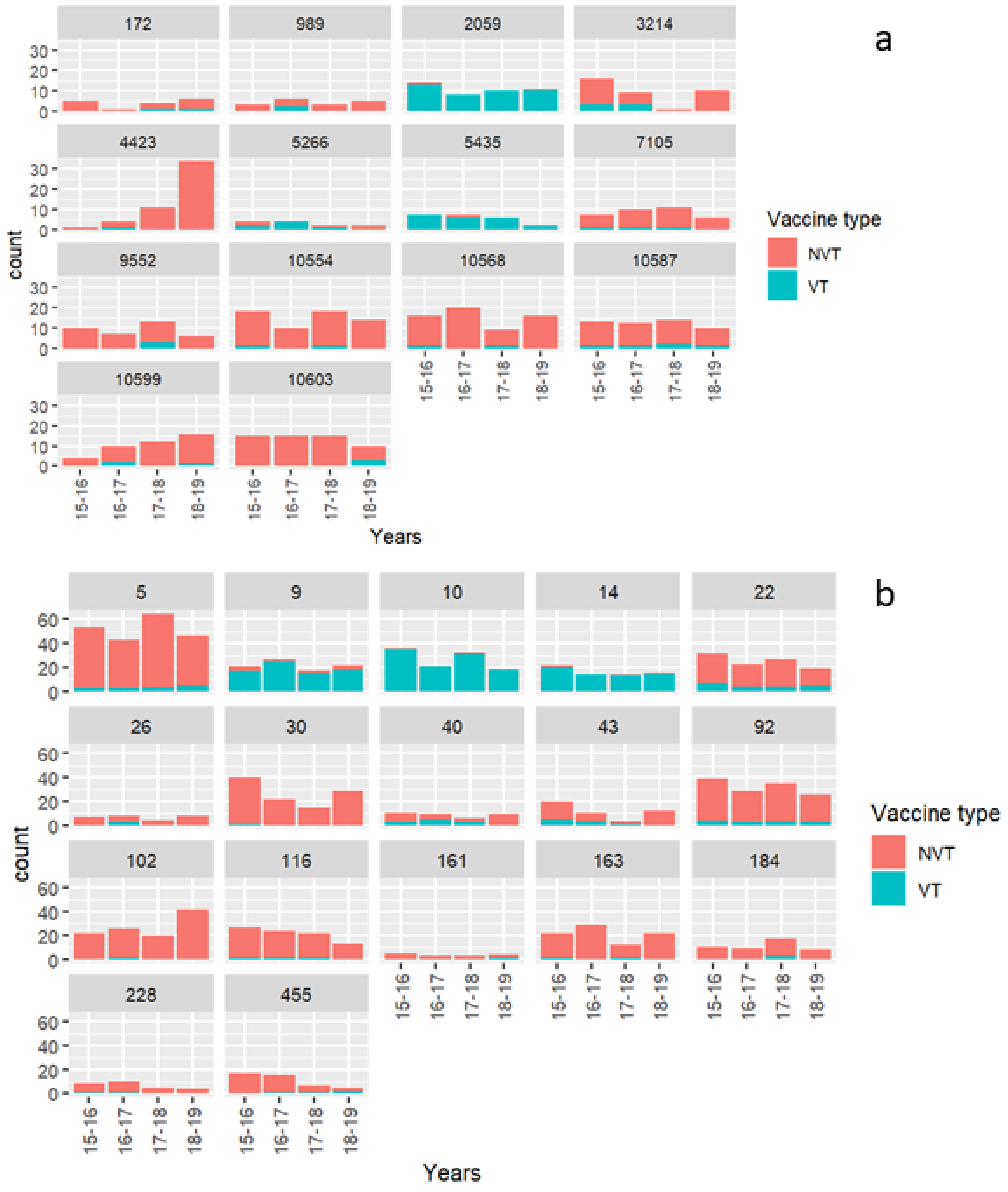

Figure S3 – (a) metabolic genotypes and (b) serotypes significantly different in distribution between survey 1-4 and survey 5-8. Statistical association between MT (c) or serotype (d) and penicillin resistance. (e) Penicillin resistance MIC in strains isolated during the early (survey 1-4) or late (survey 5-8) stages of the carriage surveys.

Panels a, b, c and d show volcano plots based on the p-value and the odds-ratio of fisher's exact tests, calculating the significance of: each MT being differently distributed in survey 1-4 vs. 5-8 (a); each serotype being differently distributed in survey 1-4 vs. 5-8 (b); each strain in a MT showing a penicillin MIC higher than 0·06 ug/ml (c); each strain in a serotype showing a penicillin MIC higher than 0·06 ug/ml (d). Yellow and red lines show a significance of <0·01 (red) <0·05 (yellow). Dots are colored with the same color scheme.

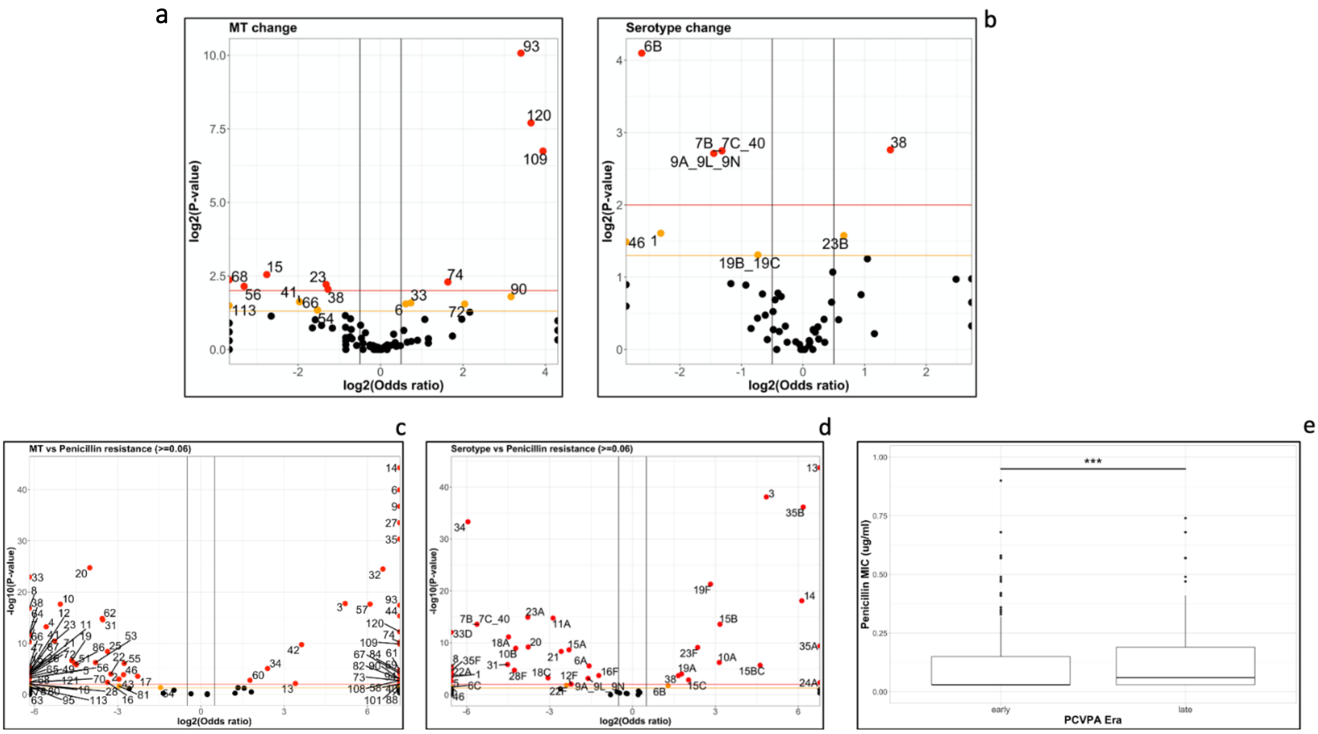

Figure S4 – Presence and absence of AMR genes in strains belonging to serotype 23B (a), 34 (b), 10A (c), 17F (d), 38 (e).

Each barplot shows the number of strains in which the specific AMR gene is present (+) or absent (-). Colors represent the metabolic genotype and are analogous to figure 3.

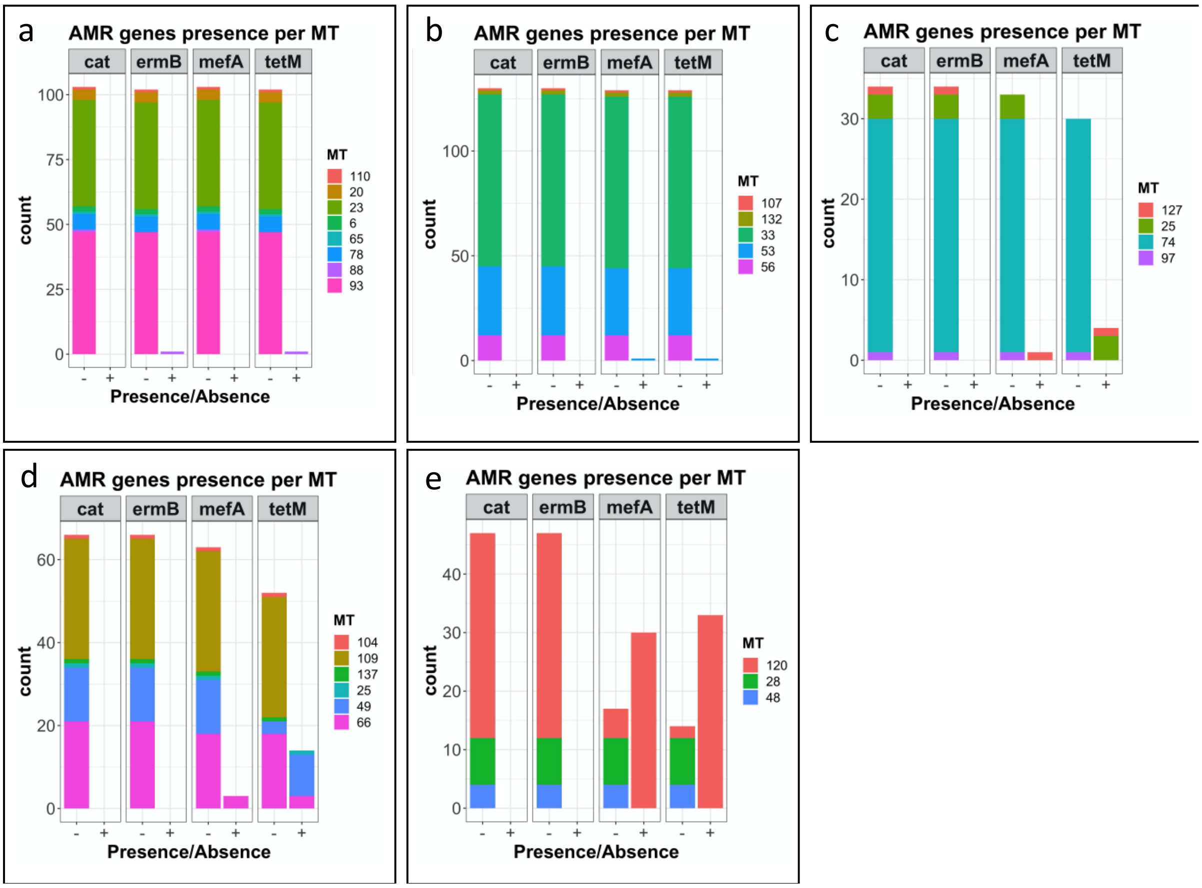

Figure S5 – Frequency of MTs, penicillin MIC and frequency of AMR genes in serotype 3 and 23F, during the 8 carriage surveys.

Each panel shows the frequency of isolation of each metabolic profile (connected points), penicillin MIC of each isolate (box and whiskers plot, with points representing each isolate), and presence of AMR genes in each metabolic type (regardless of isolation time - barplot). Panels correspond to serotypes 3 (top) and 23F (bottom). The box and whiskers plot in each panel also shows the number of isolates per survey at the top. Vertical blue lines separate the early-late isolates.

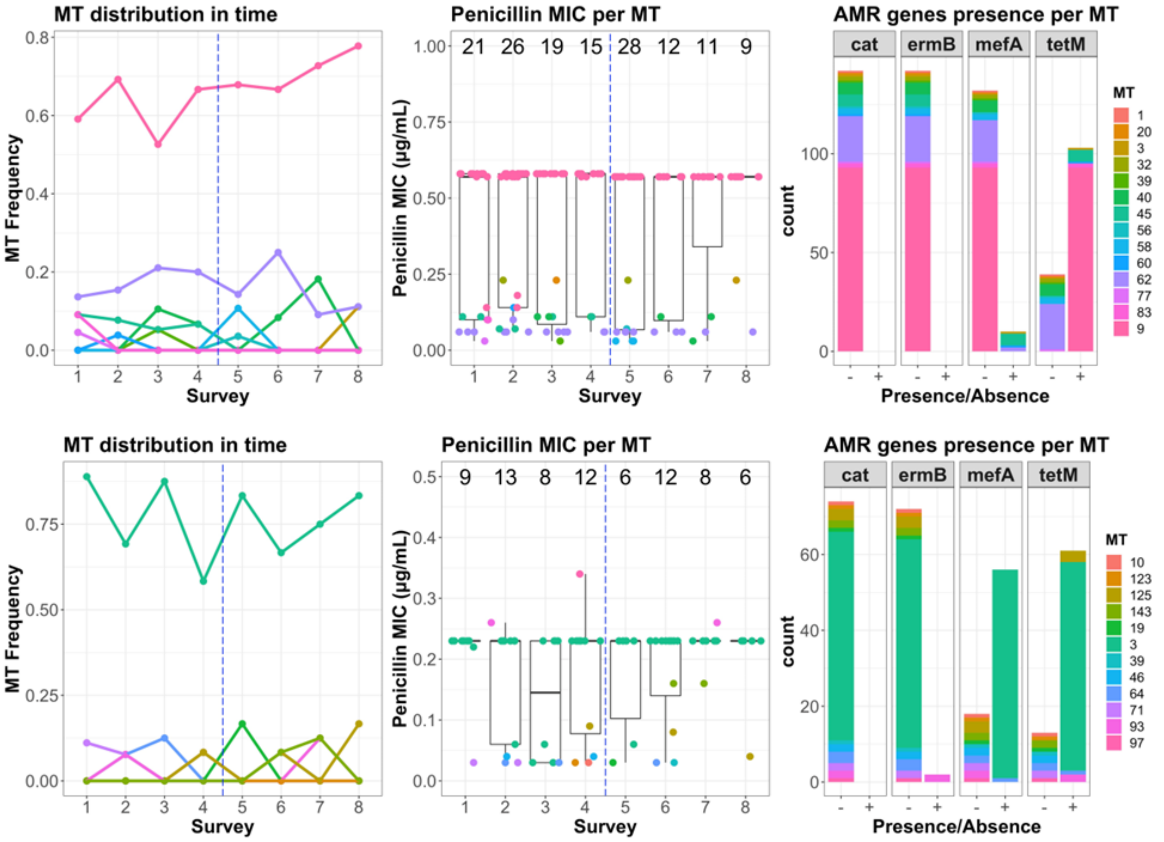

Figure S6 - Core genome SNPs count for serotypes 38 (a), 17F (b), 34 (c), 10A (d), 23B (e).

Core genome SNPs were normalised against the lower SNP count in the core-genome alignment in each genotype (* F-test for equality of variances, p-value < 0·05).

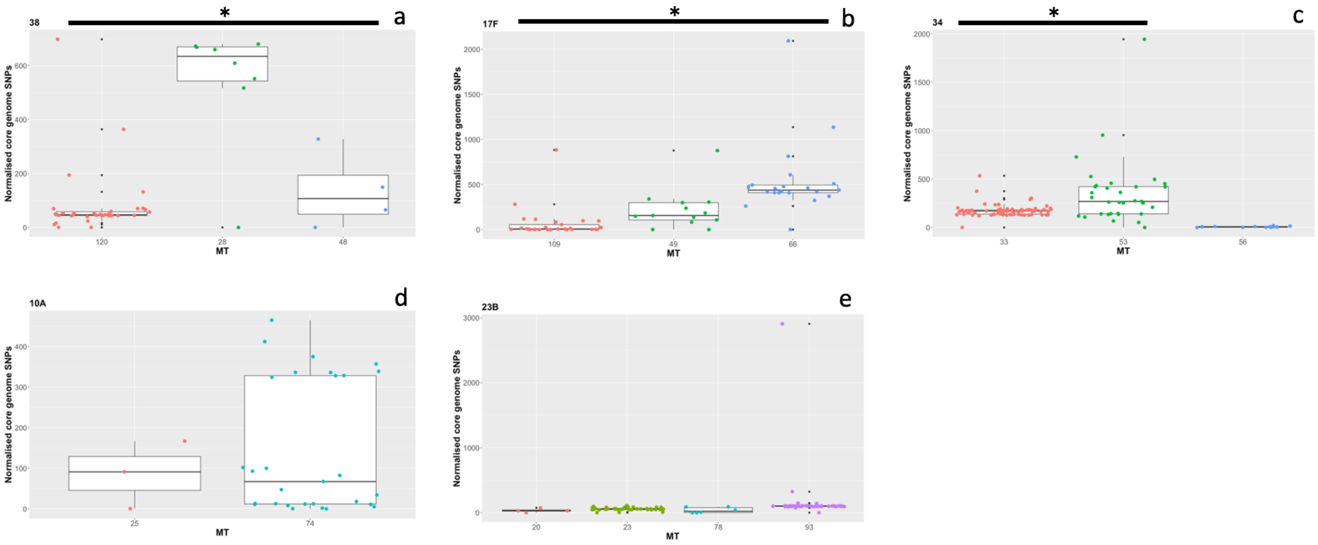

Figure S7 – Root-to-tip regression for strains isolated between 2015 and 2019 in Blantyre in the context of this study, for serotypes 38 (a) and 17F (b).

Dots on the tree are coloured from blue to red, according to their isolation date reflecting the dots on the linear regression plot. Nodes 85, 84 and 83 are highlighted in (a); Nodes 94 and node 95 are highlighted in (b).

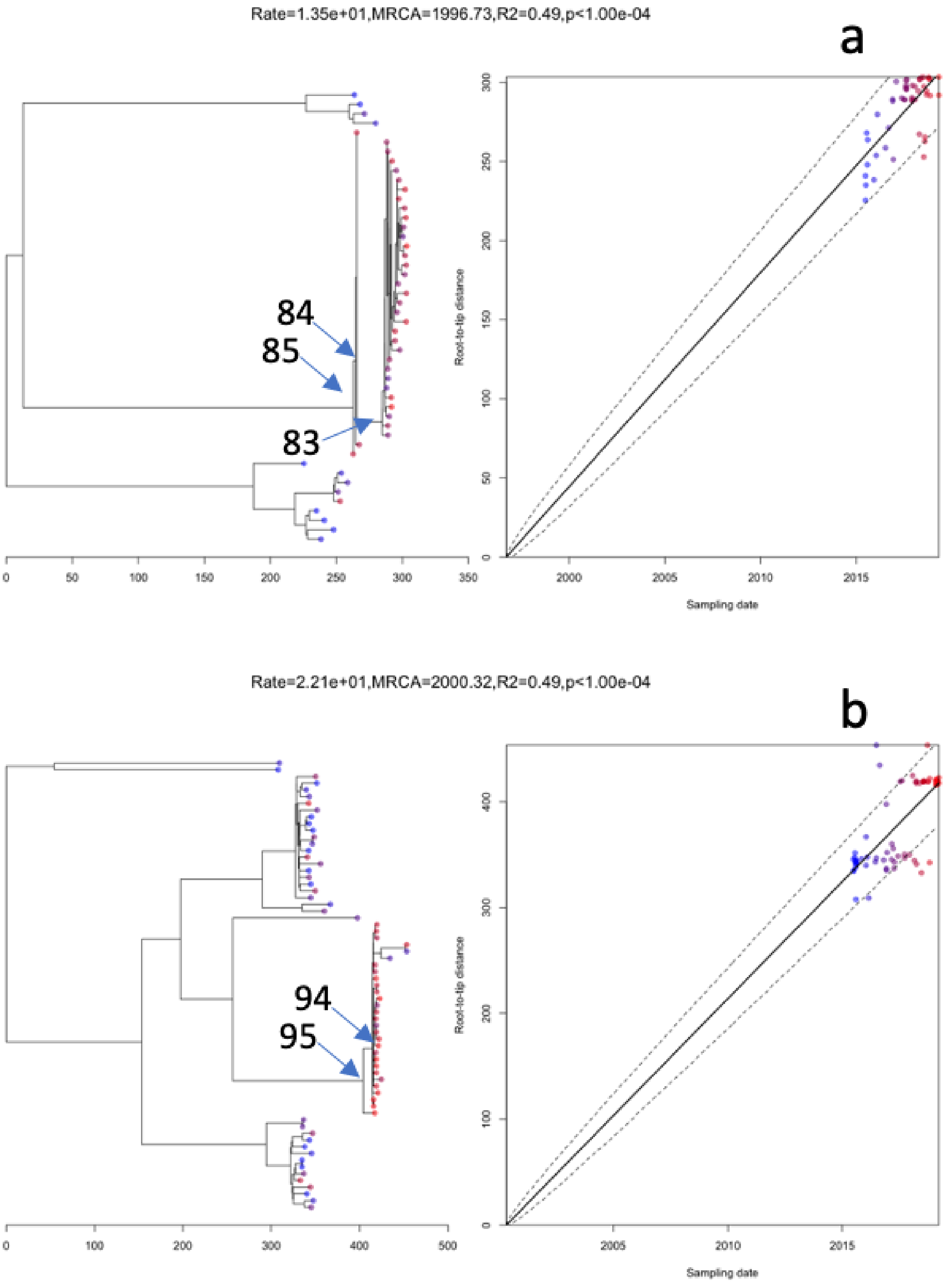

Figure S8 - Phylogenetic trees for post-vaccine carriage isolates of serotype (a) 10A, (b) 17F, (c) 38, (d) 23B, (e) 34.

Each tree is annotated with the metabolic genotypes (MT, colored strip) and with a binary heatmap showing gene presence (Green) or absence (Red) for the typical genes identified in the most common metabolic profile in the late stages of carriage surveys.

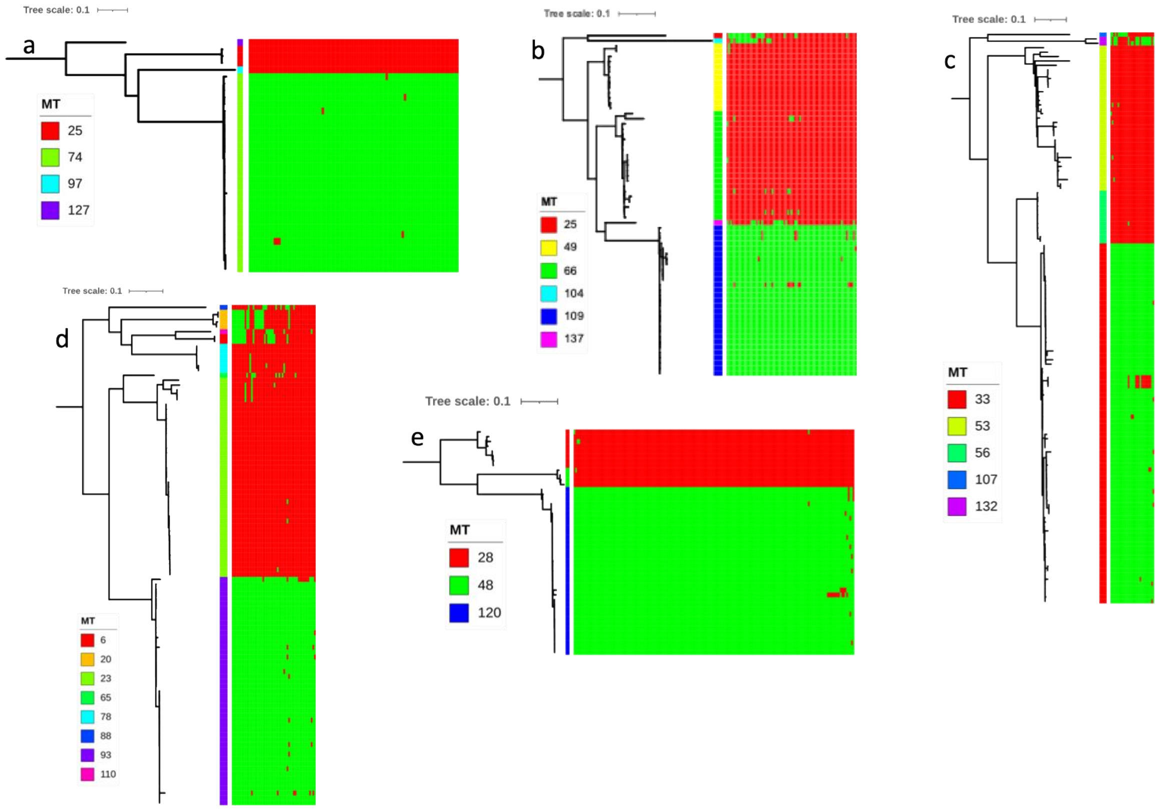

Figure S9 - Core genome SNPs count for serotype 38 – MT 120 strains isolated in Malawi and in South Africa.

Core genome SNPs were normalised against the lower SNP count in the core-genome alignment in each serotype [* F-test for equality of 2 variances, p-value < 0·05]. In the lower plot, SNP count is normalised against the lowest SNP value in each group (Malawi and South Africa).

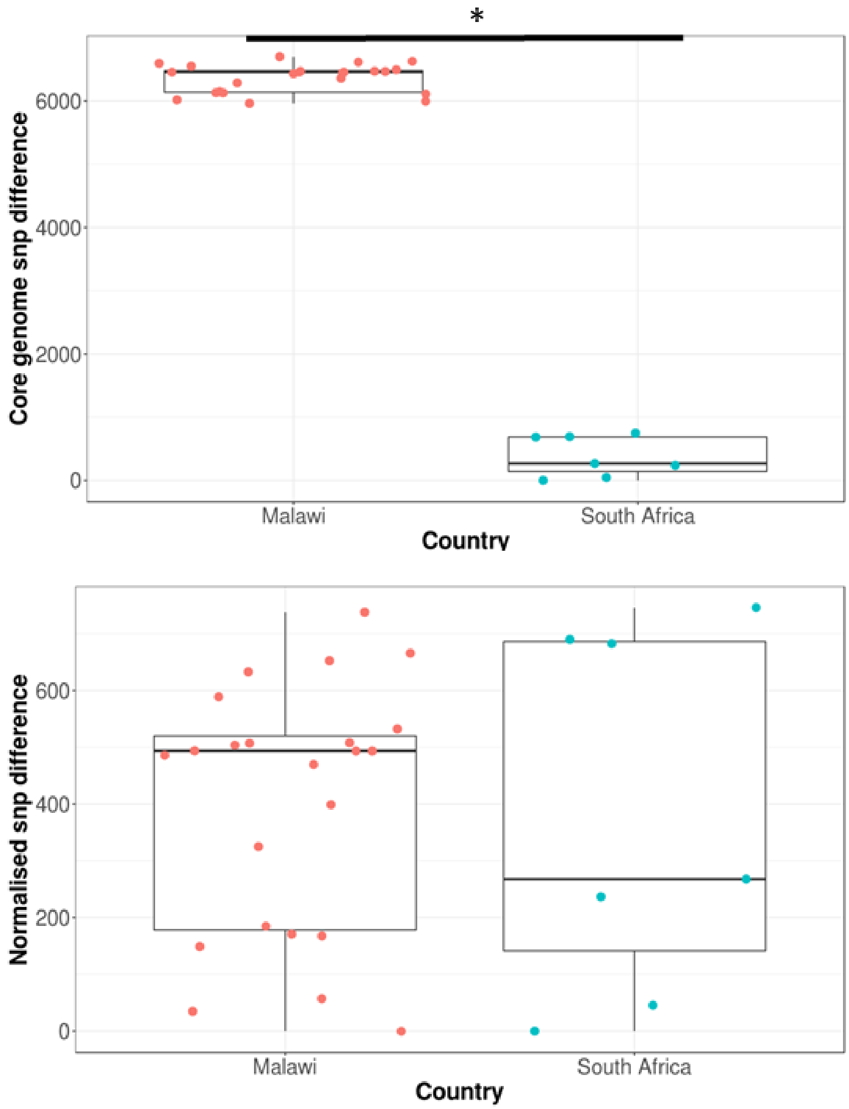

Figure S10 – Growth curves and hemolysis patterns for MT9 and MT60 (serotype 3) and serotype MT3 and MT123 (serotype 23F).

24-hour growth curves at 37°C, 5% CO_2 for serotype 3 MT9 and MT60 (A) and serotype 23F MT3 and MT123 (B). (C) Hemolysis patterns promoted by MT9 and MT60 (serotype 3) and serotype MT3 and MT123 (serotype 23F) were determined for matched CFU-pairs. Graphs show the average results from 3 independent experiments. Statistical significance was determined using Student's t-test assuming equal variance.

A.

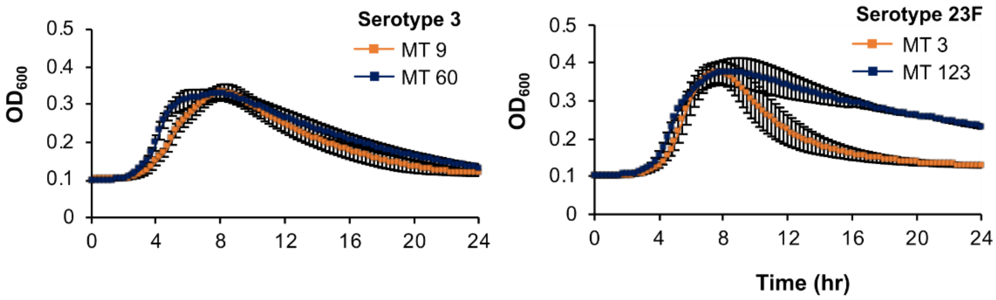

B.

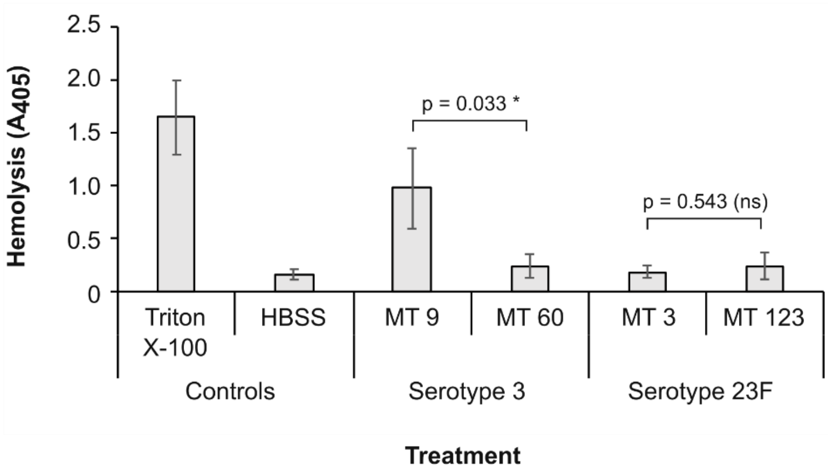

Figure S11 – linkage disequilibrium.

Linkage disequilibrium scores for metabolic (red) and non-metabolic (blue) genes for 100 randomly selected pairs (a) and for all genes (b). D’ scores are plotted in (a) over genetic distance, determined by the ATCC 700669 reference genome. The difference between D’ scores is not statistically significant in (b) (Wilcoxon ranked-sum test, p-value=0.19).

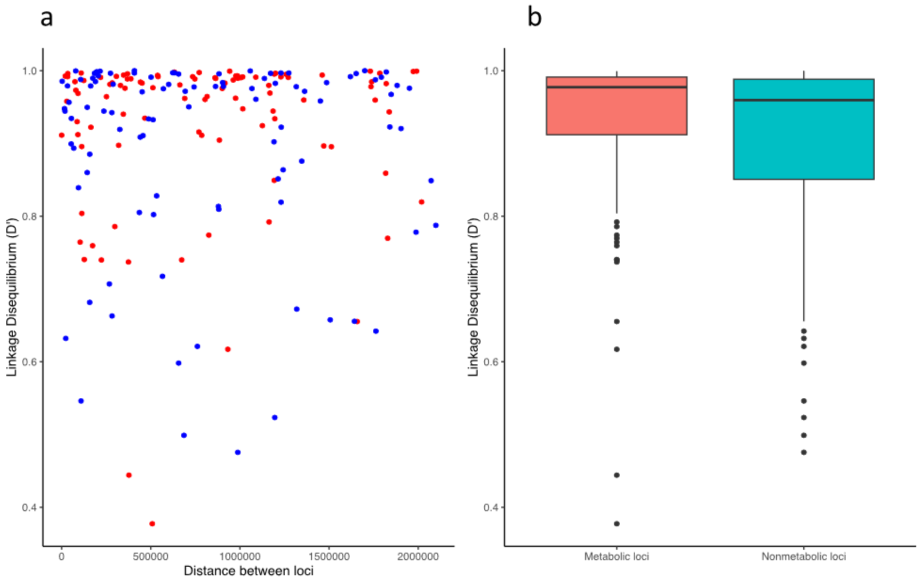

Supplement: Supplementary file 1 — Supplementary Information [file 41467_2023_43160_MOESM1_ESM.pdf]
