# Supplementary material for: The metabolic, virulence and antimicrobial resistance profiles of colonising Streptococcus pneumoniae shift after PCV13 introduction in urban Malawi
Source: Nat Commun. 2023 Nov 17;14:7477. doi: 10.1038/s41467-023-43160-y (PMC10656543; doi:10.1038/s41467-023-43160-y)
Supplement: Supplementary file 2 — Description of Additional Supplementary Files [file 41467_2023_43160_MOESM2_ESM.pdf]

Description of Additional Supplementary Files

Supplementary Data 1 – GWAS analysis results

For each serotype, the genes significatively enriched in the dominant MT are reported. Hypothetical proteins (as identified by Prokka automated annotation) are excluded. All p-values are adjusted using a Bonferroni correction.

Supplementary Data 2 – Isolates sequenced in this study and associated metadata

Supplementary Data 3 – Metabolic types, and their matching GPSC and MLST

Supplementary Data 4 – Variance of virulence genes’ amino acid sequences between isolates of the same metabolic type
