# Supplementary material for: The metabolic, virulence and antimicrobial resistance profiles of colonising Streptococcus pneumoniae shift after PCV13 introduction in urban Malawi
Source: Nat Commun. 2023 Nov 17;14:7477. doi: 10.1038/s41467-023-43160-y (PMC10656543; doi:10.1038/s41467-023-43160-y)
Supplement: Supplementary file 7 — Reporting Summary [file 41467_2023_43160_MOESM7_ESM.pdf]

nature portfolio

Corresponding author(s): Robert Heyderman

Last updated by author(s): Sep 15, 2023

Reporting Summary

Nature Portfolio wishes to improve the reproducibility of the work that we publish. This form provides structure for consistency and transparency in reporting. For further information on Nature Portfolio policies, see our Editorial Policies and the Editorial Policy Checklist.

Statistics

For all statistical analyses, confirm that the following items are present in the figure legend, table legend, main text, or Methods section.

n/a	Confirmed	
☐	☒	The exact sample size (*n*) for each experimental group/condition, given as a discrete number and unit of measurement
☐	☒	A statement on whether measurements were taken from distinct samples or whether the same sample was measured repeatedly
☐	☒	The statistical test(s) used AND whether they are one- or two-sided<br>*Only common tests should be described solely by name; describe more complex techniques in the Methods section.*
☐	☒	A description of all covariates tested
☐	☒	A description of any assumptions or corrections, such as tests of normality and adjustment for multiple comparisons
☐	☒	A full description of the statistical parameters including central tendency (e.g. means) or other basic estimates (e.g. regression coefficient) AND variation (e.g. standard deviation) or associated estimates of uncertainty (e.g. confidence intervals)
☐	☒	For null hypothesis testing, the test statistic (e.g. *F*, *t*, *r*) with confidence intervals, effect sizes, degrees of freedom and *P* value noted<br>*Give P values as exact values whenever suitable.*
☒	☐	For Bayesian analysis, information on the choice of priors and Markov chain Monte Carlo settings
☒	☐	For hierarchical and complex designs, identification of the appropriate level for tests and full reporting of outcomes
☒	☐	Estimates of effect sizes (e.g. Cohen's *d*, Pearson's *r*), indicating how they were calculated

Our web collection on statistics for biologists contains articles on many of the points above.

Software and code

Policy information about availability of computer code

Data collection	All software used in this study is explicitly detailed in the manuscript and the supplementary files
Data analysis	All data analysis protocols are explicitly detailed in the manuscript and the supplementary files

For manuscripts utilizing custom algorithms or software that are central to the research but not yet described in published literature, software must be made available to editors and reviewers. We strongly encourage code deposition in a community repository (e.g. GitHub). See the Nature Portfolio guidelines for submitting code & software for further information.

Data

Policy information about availability of data

All manuscripts must include a data availability statement. This statement should provide the following information, where applicable:

- Accession codes, unique identifiers, or web links for publicly available datasets
- A description of any restrictions on data availability
- For clinical datasets or third party data, please ensure that the statement adheres to our policy

The data underlying the findings of the study are available either as supplementary files or archived at https://pubmlst.org/organisms/streptococcus-pneumoniae, as detailed in the manuscript. All isolate sequences are available from NCBI (https://www.ncbi.nlm.nih.gov/bioproject/PRJNA1011974).

Research involving human participants, their data, or biological material

Policy information about studies with human participants or human data. See also policy information about sex, gender (identity/presentation), and sexual orientation and race, ethnicity and racism.

Reporting on sex and gender	Not relevant to this study
Reporting on race, ethnicity, or other socially relevant groupings	Not relevant to this study
Population characteristics	As reported in Swarthout 2020:<br>This observational filed-based study occurred in medium- and high-density townships of urban Blantyre, Malawi. We recruited:<br>i) healthy (reported) children aged 4 weeks to 10 years, regardless of gender (50.3& male [2708/5378] & 49.7% female (2670/5378) and<br>ii) HIV-infected adults 18-40 years old and on antiretrovial therapy (ART), regardless of gender (68% male [1141/1770] & 32% female [559/1770]).
Recruitment	As reported in Swarthout 2020:<br>Participation was voluntary, though participation of screened individuals was high. For children recruited from schools, letters were sent home inviting parents/guardians to travel to the school to discuss the study and consider consenting to their child's participation. If the parent/guardian did not respond to the letter or did not visit the school within the specified 3 days, another child was randomly selected from the school's register. The number of letters actually received by the parents/guardians and the reasons for parents/guardians not accepting the invitations were not routinely documented. During household recruitment, study teams maintained a diary to record the number of homes visited. An average of 7·2 households were approached for every child screened. HIV-infected adults 18–40 years old and on ART were recruited from Blantyre's Queen Elizabeth Central Hospital ART Clinic using systematic sampling. Though difficult to determine, it is not expected that any self-selection would significantly influence the study findings.
Ethics oversight	The study protocol was approved by the College of Medicine Research and Ethics Committee, University of Malawi (P.02/15/1677) and the Liverpool School of Tropical Medicine Research Ethics Committee (14.056).

Note that full information on the approval of the study protocol must also be provided in the manuscript.

Field-specific reporting

Please select the one below that is the best fit for your research. If you are not sure, read the appropriate sections before making your selection.

☒ Life sciences ☐ Behavioural & social sciences ☐ Ecological, evolutionary & environmental sciences

For a reference copy of the document with all sections, see nature.com/documents/nr-reporting-summary-flat.pdf

Life sciences study design

All studies must disclose on these points even when the disclosure is negative.

Sample size	The observational part of the study relied on the data collected for another study (Swathout et al. Nature communications, 2021). There, the sample size strategy was pragmatic to allow for adequate precision of the carriage prevalence estimates. The sample size determined for the colonization experiments was based on logistical constraints.<br>The number of biological replicates performed for mice, bacterial growth and hemolysis assays (n=3-5) were in line with standards in the field and allow for statistical testing using standard parametric statistical tests. No sample size calculations were performed for these experiments.
Data exclusions	No data were excluded.
Replication	All the experimental settings used in the study are sufficiently detailed to facilitate replication.
Randomization	The observational part of the study follow Swarthout et a. in which nfants 4-8 weeks were recruited from vaccination centres using systematic sampling. Children 18 weeks–7 years were recruited from households within pre-defined clusters. Both cluster and household were randomly selected. Within each cluster, after randomly choosing a first house using a 'spin the bottle' technique, teams moved systematically, recruiting one eligible child per household until the required number of children were recruited from each cluster. Children 5-10 years old were recruited from schools. A computer-generated randomized number list was used to select children from the school register. HIV-infected adults 18–40 years old and on ART, were recruited from Blantyre's Queen Elizabeth Central Hospital ART Clinic using systematic sampling.<br>For the experimental results, mice were randomly allocated to colonization with different strain pf pneumococci.<br>Allocation into experimental groups and control of covariates were not applicable for mice, bacterial growth and hemolysis assays, as individual strains were treated independently without grouping.
Blinding	The only blinding that occurred was in blinding the laboratory technicians to the source of collected samples.

Reporting for specific materials, systems and methods

We require information from authors about some types of materials, experimental systems and methods used in many studies. Here, indicate whether each material, system or method listed is relevant to your study. If you are not sure if a list item applies to your research, read the appropriate section before selecting a response.

Materials & experimental systems

n/a	Involved in the study	
☒	☐	Antibodies
☐	☒	Eukaryotic cell lines
☒	☐	Palaeontology and archaeology
☐	☒	Animals and other organisms
☒	☐	Clinical data
☒	☐	Dual use research of concern
☒	☐	Plants

Methods

n/a	Involved in the study	
☒	☐	ChIP-seq
☒	☐	Flow cytometry
☒	☐	MRI-based neuroimaging

Eukaryotic cell lines

Policy information about cell lines and Sex and Gender in Research

Cell line source(s)	Human pharyngeal carcinoma Detroit 562 epithelial cells (ATCC_CCL-138).
Authentication	Purchased directly from ATCC so no personal authentication was conducted.
Mycoplasma contamination	All cell lines were tested for mycoplasma by PCR on a monthly basis and were continuously negative.
Commonly misidentified lines (See ICLAC register)	N/A

Animals and other research organisms

Policy information about studies involving animals; ARRIVE guidelines recommended for reporting animal research, and Sex and Gender in Research

Laboratory animals	mus musculus, outbred CD1 mouse, 5-6 weeks old.
Wild animals	The study did not involve wild animals.
Reporting on sex	The study only involved the use of female mice (n=30) because of a previously demonstrated sex-based difference in susceptibility to respiratory and systemic pneumococcal disease (Kadioglu et al, The Journal of Infectious Diseases, 2011).
Field-collected samples	The study did not involve samples collected from the field.
Ethics oversight	All animal procedures were approved by the local (UCL) ethical review process and conducted in accordance with the relevant, UK Home Office–approved, project license (PPL70/6510).

Note that full information on the approval of the study protocol must also be provided in the manuscript.
